# Ubiquitin-specific protease 26 facilitates endochondral ossification by driving chondrocyte hypertrophy and mineralization

**DOI:** 10.1038/s41413-026-00517-5

**Published:** 2026-04-09

**Authors:** Changwei Li, Yiming Xu, Li Zhou, Leilei Chang, Zhou Dan, Yunhe Jiang, Chao Wang, Lianfu Deng, Guoqing Tang

**Affiliations:** 1 Department of Orthopedics, Shanghai Key Laboratory for Prevention and Treatment of Bone and Joint Diseases, Shanghai Institute of Traumatology and Orthopedics, Ruijin Hospital, Shanghai Jiao Tong University School of Medicine, Shanghai, China; 2 Department of Orthopedics, Kunshan Hospital of Chinese Medicine, Affiliated Hospital of Yangzhou University, Suzhou, Jiangsu Province China; 3 Institute of Traumatology and Orthopedics, Kunshan Hospital of Chinese Medicine, Affiliated Hospital of Yangzhou University, Suzhou, Jiangsu Province China; 4 Department of Orthopedics, The Shanghai Tenth People’s Hospital of Tongji University, Shanghai, China

**Keywords:** Bone, Energy metabolism, Osteoporosis

## Abstract

Chondrocyte hypertrophy and mineralization are essential for endochondral ossification; however, the mechanisms underlying these processes remain incompletely understood. In this study, we have identified the facilitated role of ubiquitin-specific protease 26 (USP26) in endochondral ossification by stimulating chondrocyte hypertrophy and mineralization. Ultimately, this promotes skeletal development, bone fracture healing, and the occurrence of osteoarthritis. Mechanistically, USP26 decreases FBP2 undergoing K63-linked ubiquitination, leading to a reduction in the protein level of FBP2. This reduction promotes mitochondrial biogenesis and oxidative phosphorylation, thus facilitating chondrocyte hypertrophy and mineralization and aiding in the process of endochondral ossification. Furthermore, our study found that compression loading induces USP26 to initiate chondrocyte hypertrophy and mineralization through the phosphorylation of estrogen receptor-α at serine 118. These findings suggest that USP26, acting as a mechanosensor, facilitates chondrocyte hypertrophy and mineralization by maintaining mitochondrial biogenesis through the reduction of FBP2. Identifying USP26 as a potential therapeutic target for physiological skeletal growth, bone fracture healing, and osteoarthritis.

## Introduction

Endochondral ossification is not only necessary for physiological skeletal growth, but also plays a crucial role in the development of bone fracture healing and osteoarthritis (OA).^1–4^ The process of endochondral ossification requires chondrocytes to undergo hypertrophic differentiation, a stage characterized by the expression of type X collagen (COL10A1). Chondrocyte hypertrophy promotes angiogenesis and mineralization, ultimately resulting in the transformation of avascular cartilage into highly vascularized bone tissue.^1,3,5^ Matrix metalloproteinase 13 (MMP13) plays a significant role in matrix degradation^3,6^ while vascular endothelial growth factor (VEGF) triggers an angiogenic switch that facilitates vascular invasion.^3,7^

Although the exact mechanisms underlying chondrocyte hypertrophy and mineralization remain unclear, it is possible that certain pathways related to bone formation are involved in the process. In this process, the transforming growth factor-beta (TGF-β) family of growth factors,^8^ HIF-2α transcriptional factor,^3^ Indian hedgehog (Ihh),^9^ parathyroid hormone–related peptide (PTHrP/PTH),^10^ Wnt/β-catenin,^11^ and RBPjκ-dependent Notch^4^ signaling may play important roles. Recently, it has been discovered that the ubiquitin-specific protease 26 (USP26) plays a crucial role in promoting osteogenic differentiation of bone marrow mesenchymal stem cells (BMSCs) and osteoblasts.^12–14^ Knocking out USP26 significantly reduces the expression of key genes necessary for bone formation, such as runt-related transcription factor 2 (Runx2), osterix, osteocalcin (Ocn), alkaline phosphatase (Alp), and bone morphogenetic protein-2 (Bmp2), leading to a significant decrease in bone mass.^12–14^ However, whether USP26 can regulate endochondral ossification, especially via its effects on chondrocyte hypertrophy and mineralization, remains unclear.

In the present study, we have discovered that USP26 can participate in endochondral ossification by driving chondrocyte hypertrophy and mineralization as a mechanosensor. This process is achieved by supporting glucose consumption through the reduction of FBP2. Identifying USP26 as a potential therapeutic target for physiological skeletal growth, bone fracture healing, and osteoarthritis.

## Results

### USP26 is increased during endochondral ossification

**Fig. 1:**
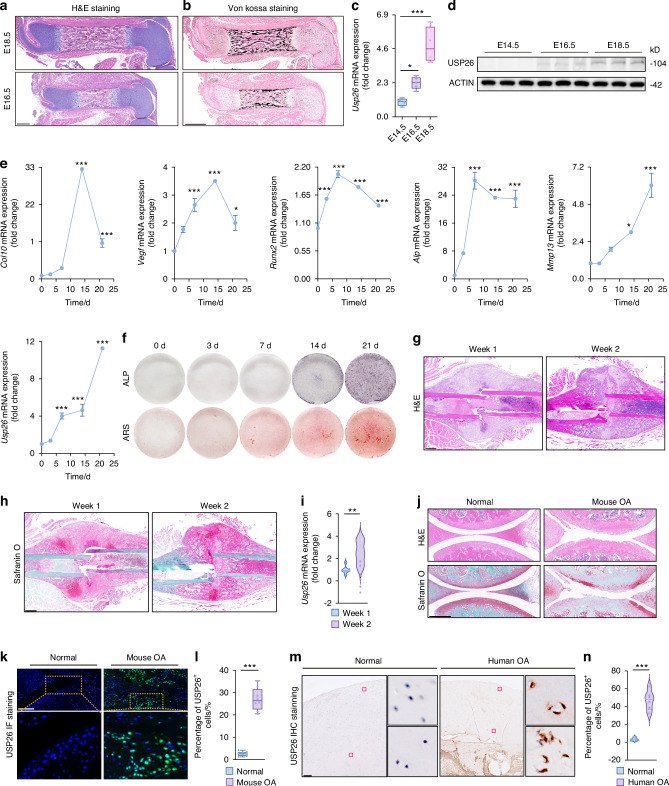
USP26 is increased during chondrocyte differentiation and endochondral ossification. **a**, **b** H&E and Von Kossa staining were performed on whole femurs at E16.5 and E18.5. Scale bars represent 300 μm. **c**, **d** The mRNA expression and protein levels of USP26 were analyzed in femurs at E14.5, E16.5, and E18.5. **e**, **f** The mRNA expression levels of Col10, Mmp13, Vegf, Runx2, and Alp, as well as ALP and ARS staining, were examined in chondrocytes undergoing differentiation over 0, 3, 7, 14, and 21 days. **g**, **h** H&E and Safranin O-Fast Green staining were performed on the callus at 1 and 2 weeks post-femur fracture. Scale bars represent 500 μm. **i** The mRNA expression of USP26 in the femur callus was evaluated at 1 and 2 weeks following fracture. **j**–**l** H&E staining, Safranin O-Fast Green staining, and USP26 immunofluorescence staining were performed on the knee joints of mice in both the surgical OA group and the control group. Black and white scale bars represent 300 μm and 150 μm, respectively. **m**, **n** Immunohistochemical staining for USP26 in the knee joint cartilage of normal and osteoarthritis (OA) patients was conducted. Scale bars represent 900 μm. ^*^P < *0.05*, ^**^P < *0.01*, ^***^P < *0.001*. *P*-values were analyzed by one-way ANOVA in **c**, **e** and two-tailed *t* tests in **i**, **l**, **n**

In mice, limb mesenchymal condensation occurs at around embryonic day 11.5–12.5 (E11.5–E12.5).^1^ After condensation, the majority of mesenchymal progenitors, which express SOX9, become chondrocytes that undergo the differentiation and maturation program during embryonic development.^1^ As shown in Fig. 1a, b, a primary ossification center (POC) had formed in the middle of the femur at E16.5. By E18.5, the size of the POC area had further increased, accompanied by an increase in calcium deposits. Coinciding with the occurrence of cartilage hypertrophy and endochondral ossification, there was a significant increase in both gene and protein expression of USP26 in the femur (Fig. 1c, d). To further investigate the correlation between USP26 expression and chondrocyte hypertrophy and mineralization, a differentiation induction experiment was conducted on primary articular chondrocytes. This involved using ITS for a period of 3 weeks, followed by Pi for an additional 2 days.^3,4^ During the gradual process of chondrocyte hypertrophy and mineralization, which is characterized by the expression of genes related to chondrocyte hypertrophy and osteogenic differentiation such as Collagen 10, MMP13, VEGF, RUNX2, and ALP, as well as the presence of ALP activity and calcium salt formation observed through ALP and ARS staining, the mRNA expression of USP26 was found to be increased in a time-dependent manner (Fig. 1e, f). Endochondral ossification is one of the primary methods through which fractures heal.^15^ As cartilage within the bone callus transforms into bone tissue during the healing process, the expression of USP26 in the bone callus tissue was found to significantly increase (Fig. 1g–i). During the development of osteoarthritis (OA), joint cartilage hypertrophy and ossification occur, resulting in the destruction of articular cartilage and the formation of bone spurs. This study observed a significant induction of USP26 in the joint cartilage of both osteoarthritis mouse models and patients (Fig. 1j–n). The expression of USP26 was mainly detected in highly differentiated chondrocytes within the hypertrophic zone and in the degraded cartilage of osteoarthritis patients (Fig. 1k, m). Collectively, these findings demonstrate that USP26 expression increases during chondrocyte hypertrophy, mineralization, and endochondral ossification.

### USP26 facilitates embryonic skeletal growth

**Fig. 2:**
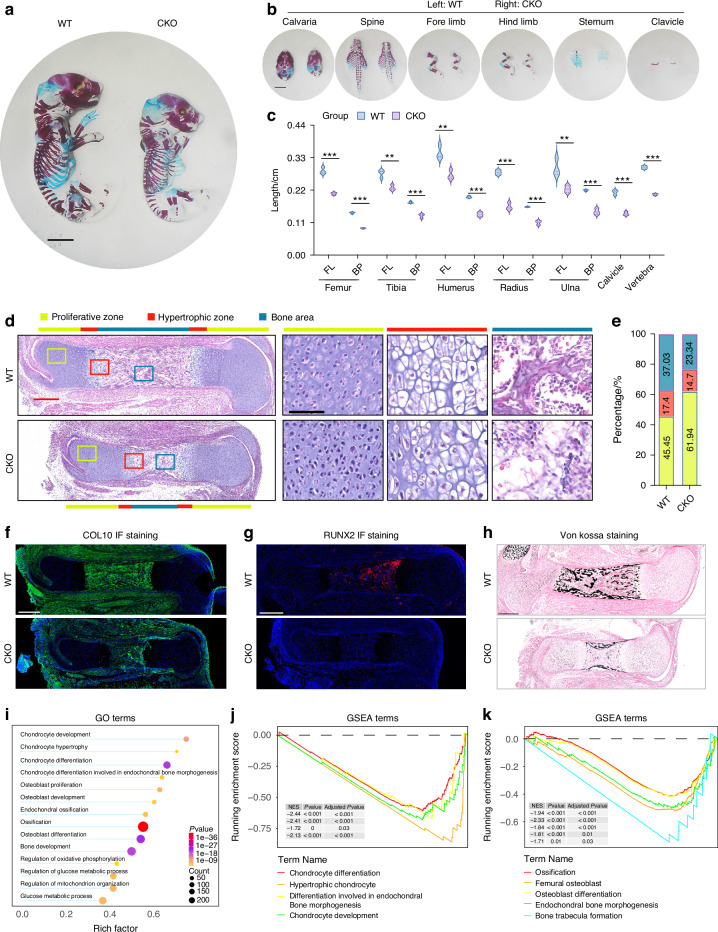
USP26 promotes chondrocyte differentiation during embryonic skeletal growth. **a**, **b** Alcian blue and alizarin red staining was performed on the whole skeleton, including the calvaria, spine, forelimbs, hindlimbs, sternum, and clavicle of Usp26 CKO mice and their littermate controls at E18.5. **c** Quantitative data from **b**, FL represents full length of the bone, which is the sum of the lengths of bones stained with Alizarin Red and bones stained with Alcian Blue. BP represents mineralized bone length, which is the length of bones stained with Alizarin Red. **d** H&E staining was performed on whole femurs of Usp26 CKO and WT littermates at E18.5. Inset boxes highlight the regions of the right three rows, representing the proliferative zone, hypertrophic zone, and bone area, indicated by yellow, red, and green bars, respectively. The red and black scale bars represent 300 μm and 50 μm, respectively. **e** Percentage of the length of the proliferative zone (yellow), hypertrophic zone (red), and bone area (green) over the total femur length of the Usp26 CKO and the WT littermate embryos was measured. **f**–**h** Immunofluorescence staining was performed on COL10 and RUNX2, as well as Von Kossa staining on full femurs of Usp26 CKO and WT littermate embryos. Scale bars represent 300 μm. **i** GO biological process analysis is utilized to identify differentially expressed genes. GSEA confirms a significant correlation between USP26 and key processes in chondrocyte differentiation (**j**) and ossification (**k**). ^**^P < *0.01*, ^***^P < *0.001*. *P*-values were analyzed by two-tailed *t* tests

To examine the physiological role of USP26 in endochondral ossification and skeletal development, we conducted a study where we conditionally inactivated USP26 in chondrocytes (Usp26^fl/fl^-Col2-Cre^+/^^−^) by intercrossing Usp26^fl/fl^ mice with Col2-Cre transgenic mice. Usp26^fl/fl^ littermates were used as the controls for this strain, referred to as Usp26^fl/fl^. Although the conditional knockout (CKO) mice developed and grew without abnormalities in major organs, they exhibited significant dwarfism during the embryonic period compared to their wild-type (WT) littermates (Usp26^fl/fl^). The limbs and vertebrae of the CKO mice were shorter than those of the WT littermates, as shown in Fig. 2a–c. Histological examination using Hematoxylin and Eosin (H&E) and Safranin O–Fast Green staining revealed that, although the actual length of the proliferative zone in the CKO limb was comparable to that of the WT controls, the percentage of the proliferative zone relative to the total limb length was slightly increased (Figs. 2d, e and S1). Furthermore, the percentage of the hypertrophic zone and bone area relative to the total limb length was considerably decreased in the CKO limbs (Fig. 2d, e). This indicates that USP26 insufficiency not only impaired chondrocyte hypertrophy but also subsequent steps such as matrix degradation and vascularization, ultimately leading to impaired endochondral ossification. Immunofluorescence staining and mRNA expression assays confirmed that COL10, MMP-13, VEGF, and RUNX2 were suppressed by USP26 insufficiency, which may explain the decrease in cartilage calcification observed in the von Kossa staining (Figs. 2f–h and S2). Additionally, apoptosis of hypertrophic chondrocytes is essential for vascular invasion, facilitating the entry of osteoclast precursors to remove cartilage and osteoblast precursors to form bone.^16^ Terminal deoxynucleotidyl transferase dUTP nick end labeling (TUNEL) staining revealed that chondrocyte apoptosis in the hypertrophic zone was significantly inhibited in Usp26 CKO mice (Fig. S3). To further investigate the regulatory role of USP26 in physiological endochondral ossification, we performed a comprehensive transcriptome analysis of femurs from *Usp26* CKO and WT littermates at E18.5. Our findings revealed that Usp26 deletion led to the downregulation of 3 542 genes and the upregulation of 3 431 genes in Usp26 CKO femurs compared to WT controls (Fig. S4A). Among the upregulated genes in Usp26 CKO femurs, we observed the presence of mediators that negatively regulate osteoblastic differentiation, such as Sost,^17^ Bambi,^18^ and Dkk1^19^ (Fig. S4B). Conversely, the expression of multiple genes that positively regulate osteoblastic differentiation, including Ihh, Bmp2, and Piezo1,^20^ was found to be decreased in Usp26 CKO femurs (Fig. S4B). Further analysis using Gene Ontology (GO) biological process analysis demonstrated a strong correlation between the differentially expressed mRNAs in WT and Usp26 CKO femurs with chondrocyte differentiation, chondrocyte hypertrophy, and endochondral ossification (Fig. 2i). Similarly, Kyoto Encyclopedia of Genes and Genomes (KEGG) pathway enrichment analysis indicated that the differentially expressed mRNAs resulting from Usp26 deletion were significantly associated with pathways related to chondrogenesis, chondrocyte hypertrophy, and endochondral ossification, including the Wnt signaling pathway, Hippo signaling pathway, and TGF-β signaling pathway (Fig. S4C). Furthermore, Gene Set Enrichment Analysis (GSEA) corroborated our findings, confirming a significant correlation between USP26 and key processes in endochondral ossification, including chondrocyte differentiation, chondrocyte hypertrophy, and endochondral ossification (Fig. 2j, k).

### USP26 benefits bone healing and stimulates OA development

**Fig. 3:**
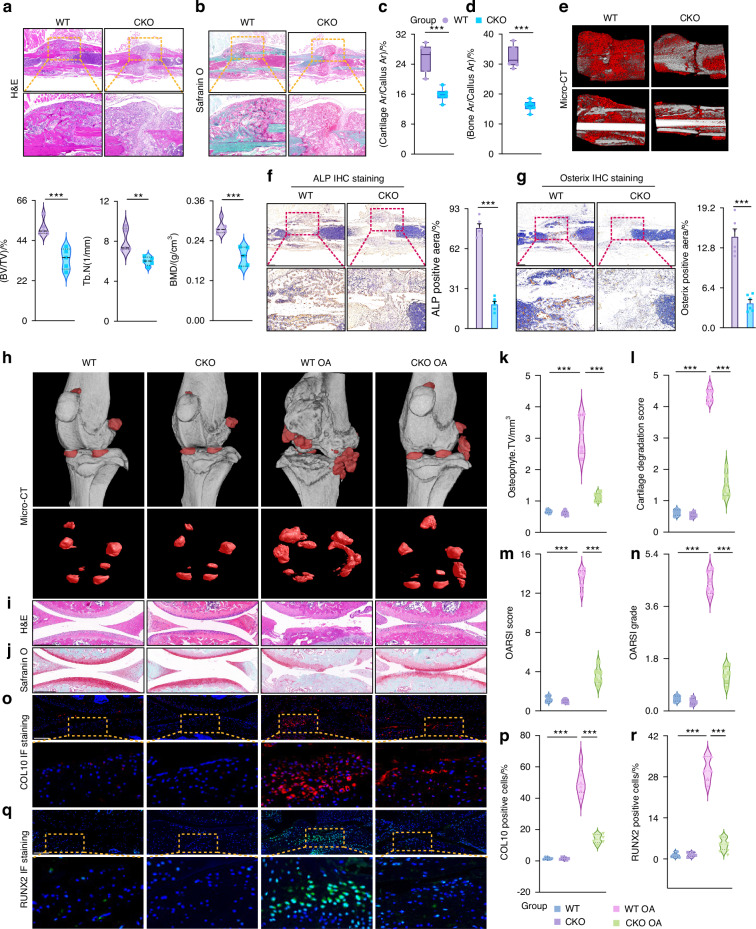
USP26 enhances chondrocyte differentiation during bone healing. **a**, **b** H&E and Safranin O-Fast Green staining were performed on the callus of Usp26 CKO mice and their WT littermates at 2 weeks post-femur fracture. Scale bars represent 500 μm. **c**, **d** Quantitative analysis was conducted to determine the cartilage and bone area as a percentage of the total callus area. **e** Representative μCT images and quantitative analysis were conducted on the bone volume fraction (BV/TV), trabecular number (Tb. N), and bone mineral density (BMD) of femur callus from Usp26 CKO mice and their WT littermates at 2 weeks post-fracture. **f**, **g** Immunohistochemical staining of ALP and Osterix in femur callus from Usp26 CKO mice and their wild-type littermates at 2 weeks post-fracture. Scale bars represent 500 μm. **h** Micro-CT scans were used to compare osteophyte formation at the cartilage periphery between Usp26 CKO mice and their WT littermates. The red regions indicate newly formed osteophytes. **i**, **j** H&E staining and Safranin O-Fast Green staining were performed on knee joints of Usp26 CKO mice and their WT littermates, both with and without surgically induced OA. Scale bars represent 300 μm. **k**–**n** Quantification of osteoarthritis development using scores for osteophyte formation, cartilage degradation, OARSI score, and OARSI grade. **o**–**r** Immunofluorescence staining was performed on knee joints from Usp26 CKO and its WT littermates, with or without surgical induction of OA, to detect COL10 and RUNX2 expression. Scale bars represent 150 μm. ^**^P < 0.01, ^***^P < 0.001. *P*-values were analyzed by two-tailed *t* tests in **c**–**g** and one-way ANOVA in **k**–**n**, **p**, **r**

Endochondral ossification is one of the main processes through which bone fractures heal.^15^ To further investigate whether Usp26 CKO impairs the healing of bone fractures, femur fractures were created as previously described.^21^ The healing process was evaluated through radiographic examination at 2 weeks post fracture, when new bone tissue was expected to be present.^22^ Serial tissue sections of the fracture callus were prepared, and the volumes of bone and cartilage within the callus were analyzed. The results shown in Fig. S5 revealed that the gene and protein expression levels of USP26 were significantly decreased in the cartilage calluses of *Usp26* CKO mice. Compared to WT mice, the volume of cartilage and bone within the fracture callus of Usp26 CKO mice was significantly smaller (Fig. 3a–d). Micro-CT results further confirmed the impaired mineralization and bone formation within the callus of the Usp26 CKO mice, as evidenced by the decreased bone volume/tissue volume (BV/TV), lower trabecular thickness (Tb. Th), trabecular number (Tb. N), and bone mineral density (BMD) (Figs. 3e and S6). Furthermore, the relative mRNA expression of Col10, Mmp13, Vegf, Runx2, and Alp determined by quantitative real-time PCR (Fig. S7), as well as the protein levels of ALP, Osterix, and OCN detected by IHC staining in the callus tissue (Fig. 3f, g and S8), further confirmed that Usp26 CKO inhibits the process of endochondral ossification during bone fracture healing.

In addition to being necessary for bone healing, chondrocyte hypertrophy and mineralization also contribute to the onset of OA.^1–4^ To investigate the involvement of USP26 in the development of OA, an experimental OA model^23^ was created in Usp26 CKO mice and their WT littermates. As shown in Fig. S9, an increase in USP26 expression was observed in the joint cartilage of OA mice. However, Usp26 CKO significantly decreased USP26 expression in the joint cartilages of both OA mice and sham controls (Fig. S9). Micro-CT analysis revealed increased formation of osteophytes in the surrounding joints of the Col2 Cre^+^-tamoxifen^-^ group mice after 2 months of OA surgery (Fig. 3h, k). Histopathological examination using H&E and Safranin O-Fast Green staining confirmed the presence of severe OA characteristics, such as surface discontinuity, vertical fissures, erosion, denudation, and deformation, in the Col2 Cre^+^-tamoxifen^-^ group mice after 2 months of OA surgery. However, these OA pathological features were significantly mitigated in the Col2 Cre^+^-tamoxifen^+^ group of mice, which had specific Usp26 deletion in chondrocytes (Fig. 3i, j, l). Moreover, the protective effects of USP26 deletion on articular cartilage degeneration were also evident in OARSI scores (Fig. 3m, n). Consistently, immunofluorescence staining revealed an increase in the expression of COL10 and RUNX2 in the joint cartilage of wild-type mice with OA (Fig. 3o–r). Conversely, the expression of these three factors was significantly reduced in the Usp26 CKO littermates. In summary, these findings demonstrate that USP26 promotes bone healing and stimulates OA development.

### USP26 stimulates chondrocyte hypertrophy and mineralization

**Fig. 4:**
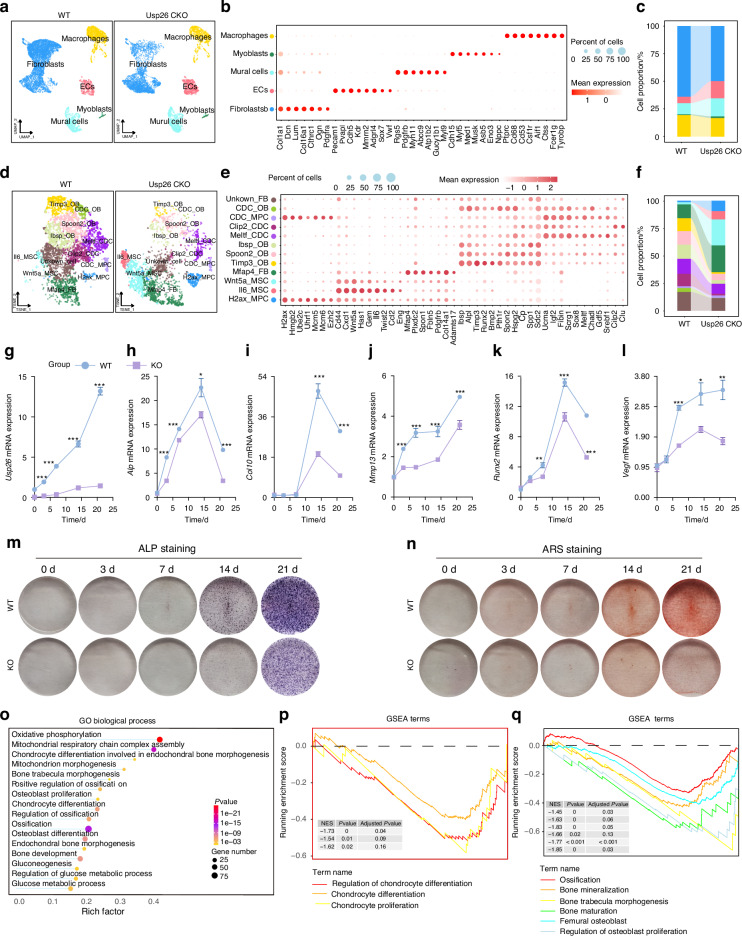
USP26 promotes chondrocyte hypertrophy and mineralization. **a** The UMAP plot pooled from five cell clusters isolated from calluses of Usp26 CKO mice and their littermate controls. **b** The dot plot illustrates the specifically expressed genes of the primary cell types, with red denoting high expression and grey indicating low expression. The circle size corresponds to the proportion of cells expressing the designated genes. **c** The proportion of each cell type in Usp26 CKO mice and their littermate controls was determined using the UMAP distribution. **d**, **e** The UMAP plot (depicted on the left) and the dot plot (displayed on the right) exhibit the re-clustered cell subgroups. Furthermore, the dot plot illustrates the specific gene expression profiles of the primary cell types extracted from the stromal cell subpopulation. In this representation, red signifies high expression levels, while grey denotes low expression. Additionally, the circle’s size reflects the proportion of cells expressing the genes indicated. **f** The stacked bar chart illustrates the proportions of each cluster separated from the fibroblast subset. **g**–**n** The mRNA expression levels of Usp26, Alp, Col10, Mmp13, Runx2 and Vegf, as well as ALP and ARS staining, were analyzed in WT and Usp26 KO chondrocytes at 0, 3, 7, 14, and 21 days post differentiation. **o** GO biological process analysis is utilized to identify differentially expressed genes. **p**, **q** GSEA confirms a significant correlation between USP26 and key processes in chondrocyte differentiation and ossification. ^*^P < 0.05, ^**^P < 0.01, ^***^P < 0.001. *P*-values were analyzed by two-way ANOVA

Chondrocyte hypertrophy and mineralization are essential processes for endochondral ossification.^5^ ‌To further explore the impact of USP26 knockout on chondrocyte evolution during endochondral ossification‌, we performed single-cell RNA sequencing (scRNA-seq) on callus tissues harvested 1 week post fracture (Fig. S10). Initial clustering analysis categorized callus cells into distinct populations: macrophages, myoblasts, mural cells, endothelial cells (ECs), and fibroblasts (Fig. 4a–c). Subsequent subclustering of fibroblasts revealed 12 transcriptional subtypes: H2ax_MPC, Il6_MSC, Wnt5a_MSC, Mfap4_FB, Timp3_OB, Spoon2_OB, Ibsp_OB, Meltf_CDC, Clip2_CDC, CDC_MPC, CDC_OB, and unknown_FB (Fig. 4d, e). Notably, the proportions of Meltf_CDC and Clip2_CDC, which represent hypertrophic chondrocytes, as well as CDC_OB, indicating chondrocytes undergoing mineralization, were all reduced in the Usp26 CKO group (Fig. 4f). These findings suggest that the deletion of Usp26 in chondrocytes stalls the process of chondrocyte hypertrophy and mineralization.

To further confirm the facilitated role of USP26 in chondrocytes hypertrophy and mineralization, primary joint articular chondrocytes isolated from Usp26 CKO mice and WT controls were induced to differentiate with ITS at various time points. Subsequently, their hypertrophy and mineralization were assessed. The results, as shown in the Fig. 4g–n, revealed that Usp26 deletion significantly impaired chondrocyte hypertrophy and mineralization. This was demonstrated by the decreased mRNA expression of Alp, Col10, Mmp13, Runx2 and Vegf (Fig. 4h–l), as well as the lower density of ALP and ARS staining (Fig. 4m, n). Additionally, the facilitated role of USP26 in the hypertrophy and mineralization was further indicated by the whole transcriptome analysis. As shown in the Fig. S11, after 21 days of differentiation induction, 1 522 genes, including Vegfa, Lgr4 and Spp1, which are positive regulators of osteogenesis, were downregulated, while 1 153 genes, including Sox9, Gli1 and Cebpd, which have been reported to inhibit osteogenic differentiation, were upregulated in Usp26^−/−^ chondrocytes compared to the WT controls (Fig. S11A, B). GO biological process analysis and GSEA revealed that the differentially expressed mRNAs were strongly correlated with chondrocyte differentiation, chondrocyte hypertrophy, and endochondral ossification (Fig. 4o–q). Correspondingly, KEGG pathway enrichment analysis revealed that multiple pathways related to chondrogenesis, chondrocyte hypertrophy, and ossification, including the Wnt signaling pathway, FoxO signaling pathway, and oxidative phosphorylation, were strongly correlated with the differentially expressed mRNAs between WT and Usp26^-/-^ chondrocytes (Fig. S11C). Collectively, these data demonstrate that USP26 promotes chondrocyte hypertrophy and mineralization.

### USP26 contributes to chondrocyte hypertrophy and mineralization by maintaining mitochondrial biogenesis

**Fig. 5:**
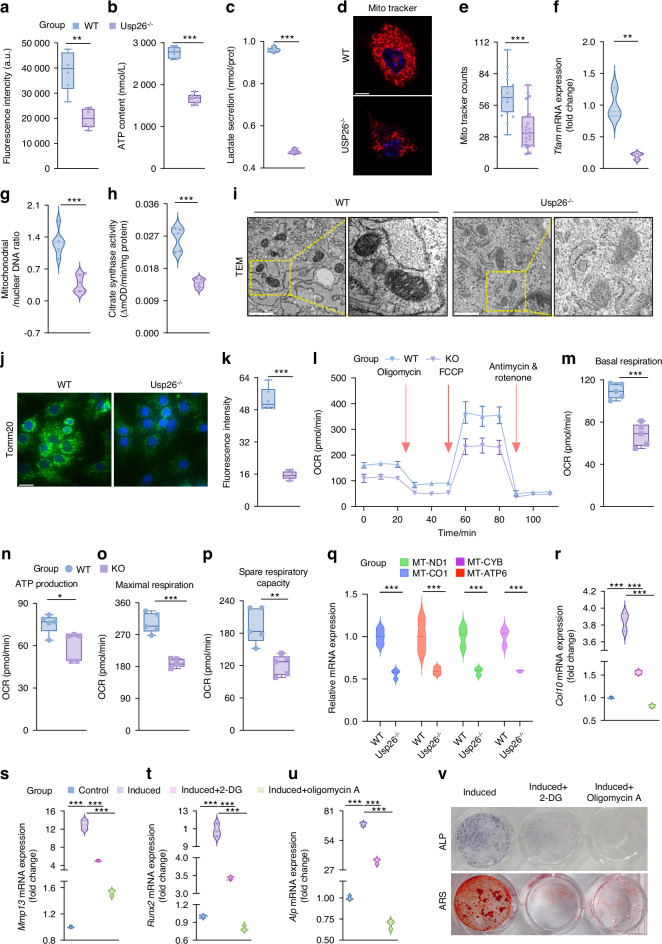
USP26 contributes to chondrocyte hypertrophy and mineralization through maintaining mitochondrial biogenesis. **a**–**c** The glucose uptake, intracellular ATP level, and lactate secretion were detected in WT and Usp26 KO chondrocytes. **d**, **e** Representative images of MitoTracker staining and quantification of MitoTracker counts of WT and Usp26 KO chondrocytes are shown. Scale bars represent 10 μm. **f** The mRNA expression of Tfam in WT and Usp26 KO chondrocytes. **g**, **h** The ratio of mitochondrial to nuclear DNA, as well as citrate synthase activity, were in WT and Usp26 KO chondrocytes. **i** Representative TEM images of mitochondria and in WT and Usp26 KO chondrocytes are shown. **j**, **k** Tomm20 immunofluorescence in WT and Usp26 KO chondrocytes. Scale bars represent 10 μm. Quantitative analysis of OCR (**l**), including basal respiration (**m**), ATP production (**n**), maximal respiration (**o**), and spare respiratory capacity (**p**). **q** The mRNA expression of MT-ND1, MT-CYB, MT-CO1, and MT-ATP6 in WT and Usp26 KO chondrocytes. **r**–**v** The mRNA expression of Col10, Mmp13, Runx2 and Alp, as well as ALP and ARS staining in chondrocytes, were assessed after 21 days of differentiation in the presence of either 2-DG or Oligomycin A. ^*^P < 0.05, ^**^P < 0.01, ^***^P < 0.001. *P*-values were analyzed by two-tailed *t* tests in **a**–**c**, **e**–**h**, **k**, **m**–**q** and one-way ANOVA in **r**–**u**

We then proceeded to determine the mechanisms by which USP26 drives chondrocyte hypertrophy and mineralization. Chondrocyte differentiation is an energy-dependent anabolic process.^24^ Based on the results of whole transcriptome sequencing, it was observed that in addition to the pathways of chondrocyte differentiation and osteogenic differentiation, glucose metabolism-related pathways, such as gluconeogenesis, oxidative phosphorylation, and mitochondrial function were also significantly enriched by the differentially expressed mRNAs between WT and Usp26^−/−^ chondrocytes, both during physiological endochondral ossification in vivo and chondrocyte hypertrophic differentiation in vitro (Figs. 2i, S4D, E, 4o and S11C). Therefore, we hypothesized that USP26 may play a role in chondrocyte hypertrophy by regulating glucose metabolism. The glucose uptake test showed that the knockout of Usp26 significantly inhibited the chondrocytes’ ability to take in glucose (Fig. 5a). Consequently, the intracellular adenosine triphosphate (ATP) level and lactate secretion experienced a significant decrease (Fig. 5b, c).

The mitochondria is the main site of cellular oxidative phosphorylation and synthesis of ATP.^25^ MitoTracker, a dye that stains active mitochondria, revealed that the normal chondrocytes’ mitochondrial network had a tubular appearance and remained intact (Fig. 5d). However, in chondrocytes with Usp26 deletion, the network was seen to be severely fragmented (Fig. 5d, e). The mRNA expression of mitochondrial transcription factor A (TFAM) and the ratio of mitochondrial to nuclear DNA were decreased in Usp26 knockout chondrocytes, indicating disrupted mitochondrial dynamics (Fig. 5f, g). Furthermore, there was a noticeable decrease in citrate synthase activity in Usp26 KO chondrocytes (Fig. 5h). Transmission electron microscopy (TEM) confirmed decreased mitochondrial biogenesis in Usp26 KO chondrocytes, as indicated by a reduced number of mitochondria and swollen mitochondria with disorganized cristae (Fig. 5i). Additionally, the expression of Tomm20, a constitutively expressed mitochondrial protein, was significantly lower in Usp26 KO chondrocytes, which further confirms the reduction in mitochondrial mass caused by USP26 knockout (Fig. 5j, k). Furthermore, the mitochondrial respiratory function, including basal respiration capacity, ATP production, maximal respiration capacity, and spare respiratory capacity (Fig. 5l–p), as well as the target genes associated with oxidative phosphorylation (OXPHOS), such as MT-ND1, MT-CYB, MT-CO1, and MT-ATP6 (Fig. 5q), were also inhibited in Usp26 KO chondrocytes. Therefore, the knockout of USP26 in chondrocytes inhibits glucose consumption by restricting mitochondrial biogenesis.

To further confirm the activation of chondrocyte hypertrophy and mineralization, which depends on glucose consumption, Oligomycin A,^26^ an inhibitor of mitochondrial ATP synthase, and FCCP,^27^ an effective uncoupler of mitochondrial oxidative phosphorylation, are being employed. The results, as shown in Fig. 5r–v, revealed that inhibition of mitochondrial function significantly impeded the hypertrophy and mineralization processes of chondrocytes.

### USP26 maintains mitochondrial biogenesis by decreasing FBP2

**Fig. 6:**
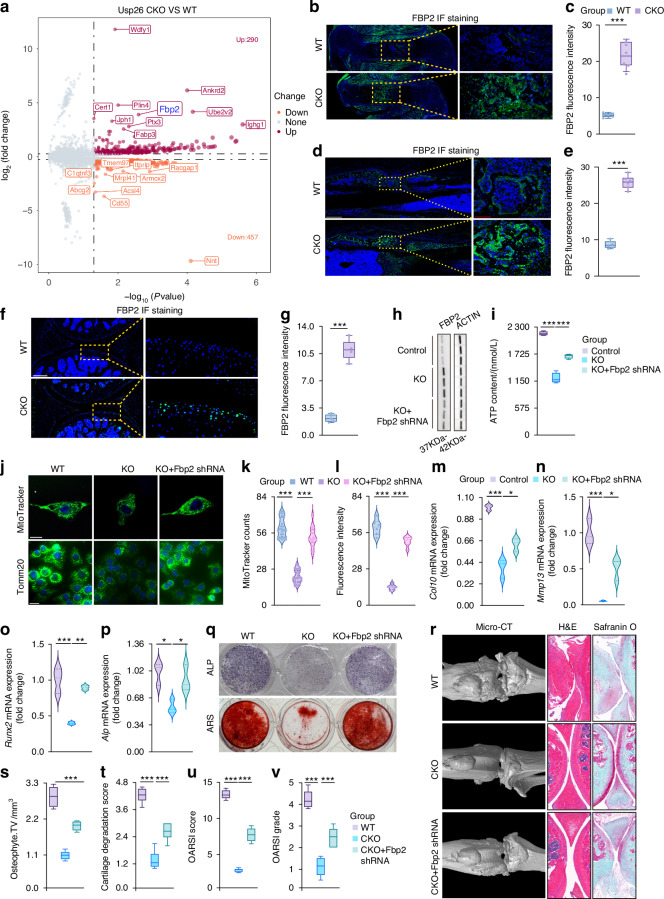
USP26 maintains mitochondrial biogenesis and chondrocyte differentiation by decreasing FBP2. **a** Proteomic sequencing was conducted on the femur of both WT and Usp26 CKO mice at E18.5. Of the proteins analyzed, 290 were found to be upregulated and 457 were downregulated. The proteins featured in the figure illustrate the top ten variances in terms of upregulation and downregulation. **b**, **c** Immunofluorescence staining for FBP2 was performed on femurs of WT and Usp26 CKO mice at E16.5. Scale bar represents 500 μm. **d**, **e** Immunofluorescence staining of FBP2 in the femoral cartilage callus following fracture. Scale bar represents 500 μm. **f**, **g** Immunofluorescence staining of FBP2 in knee joint cartilage was performed on WT and Usp26 CKO mice that were 2 months old. Scale bar represents 150 μm. **h** The protein level of FBP2 was detected by Western blot in WT and Usp26 KO chondrocytes, with and without Fbp2 knockdown. **i** The glucose uptake and intracellular ATP content were detected in WT and Usp26 KO chondrocytes, with and without Fbp2 knockdown. **j**–**l** Representative immunofluorescence staining of mitochondria and Tomm20 immunofluorescence staining in WT and Usp26 KO chondrocytes, with and without Fbp2 knockdown. The scale bar represents 1 μm in the MitoTracker staining image and 10 μm in the Tomm20 immunofluorescence staining image. **m**–**q** The mRNA expression of Col10, Mmp13, Runx2 and Alp, as well as ALP and ARS staining in WT and Usp26 KO chondrocytes with and without Fbp2 knockdown in the presence of 21 days of differentiation were analyzed. **r**–**v** Micro-CT, H&E and Safranin O-Fast Green staining were performed in knee joints to evaluate the OA progress of Usp26 CKO mice and their littermate controls, with and without Fbp2 knockdown. Scale bar represents 300 μm. ^*^P < 0.01, ^**^P < 0.01, ^***^P < 0.001. *P*-values were analyzed by two-tailed *t* tests in **c**, **e**, **g** and one-way ANOVA in **i**, **k**–**p**, **s**–**v**

USP26, as a deubiquitinating enzyme, primarily carries out its biological function by regulating protein levels.^12^ Hence, to investigate how USP26 affects the glucose consumption during chondrocyte differentiation through specific proteins, proteomic sequencing was performed on the femur of both WT and Usp26 CKO mice at E16.5. The results revealed a total of 747 differentially expressed proteins as a result of Usp26 knockout (Fig. 6a). Among these proteins, 290 were upregulated and 457 were downregulated. Some of the differentially expressed proteins, such as MMP13, Dentin matrix protein 1 (DMP1) and COL1, were found to be associated with processes like chondrocyte hypertrophy and ossification (Fig. S12A). Gene Ontology analysis confirmed that the deficiency of USP26 affects chondrocyte differentiation, endochondral ossification, and processes related to glucose metabolism, including gluconeogenesis (Fig. S12B). The fructose 1,6−bisphosphate metabolic process exhibited the highest enrichment factor (Fig. S12B). Furthermore, Kyoto Encyclopedia of Genes and Genomes (KEGG) enrichment analysis also indicated the involvement of fructose and mannose metabolism (Fig. S12C). Consistent with this, the expression of Fructose-1,6-bisphosphatase isozyme 2 (FBP2) significantly increased after USP26 knockout and ranked second among the upregulated proteins (Fig. 6a). The top-ranking protein is Ubiquitin-conjugating enzyme E2 variant 2 (Ube2v2) (Fig. 6a). FBP2 is a rate-limiting enzyme that catalyzes the irreversible hydrolysis of fructose-1,6-bisphosphate to fructose-6-phosphate and inorganic phosphate.^28^ Previous studies have reported that FBP2 inhibits glucose consumption and promotes gluconeogenesis by restraining mitochondrial biogenesis.^28^ Therefore, our hypothesis is that USP26 knockout in chondrocytes hinders glucose consumption by upregulating FBP2 expression.

Firstly, immunostaining results for FBP2 in the femoral bone tissue of E16.5 mice, the femoral cartilage callus following fracture, and the articular cartilage of the joints in 2-month-old adult mice further confirm that knockout of USP26 significantly enhances FBP2 expression in chondrocytes (Fig. 6b–g). Secondly, increased protein levels of FBP2 in Usp26^−/−^ chondrocytes was also observed in vitro through western blot detection (Fig. 6h). Additionally, the results of ATP generation (Fig. 6i), MitoTracker (Fig. 6j, k), and Tomm20 staining (Fig. 6j, l) demonstrate that the knockout of Fbp2 significantly restores the impaired glucose consumption and mitochondrial biogenesis resulting from USP26 deletion. Moreover, knockout of Fbp2 significantly rescued the impaired hypertrophic and mineralization abilities of chondrocytes caused by Usp26 deletion (Fig. 6m–q), and significantly exacerbated the pathological phenotype of OA in Usp26 CKO mice (Fig. 6r–v). Therefore, these results suggest that USP26 deletion hinders mitochondrial biogenesis and chondrocytes differentiation by increasing FBP2 expression.

To investigate whether USP26 could directly bind to FBP2 and regulate the ubiquitination degradation of FBP2, HA-tagged USP26 and Flag-tagged FBP2 were expressed in 293T cells, followed by Co-IP assays. Significantly, the presence of the HA tag was detected in anti-Flag immunoprecipitates, and vice versa (Fig. S13A, B). The overexpression of USP26 resulted in a notable decrease in the level of ubiquitinated FBP2 and led to a quicker degradation of FBP2 protein in the presence of cycloheximide (CHX) (Fig. S13C–F). Furthermore, additional studies have demonstrated that USP26 inhibits K63 (Lys63)-linked ubiquitination of FBP2. It was found that His-tagged K63-linked Ubiquitin (K63-Ub), as opposed to K48-Ub, has a stronger ability to reduce FBP2 ubiquitination in the presence of USP26 (Fig. S13E). Consistently, the direct regulatory role of USP26 in the ubiquitination and degradation of FBP2 was further verified in primary chondrocytes (Fig. S14). Given that K63-linked ubiquitin chains are known to inhibit the proteasomal degradation of cell signal regulators,^29^ it is reasonable to conclude that USP26 decreases the protein level of FBP2 by decreasing FBP2 undergoing K63-linked ubiquitination (Fig. S14F).

### Compression loading induces USP26 to initiate chondrocyte hypertrophy and mineralization

**Fig. 7:**
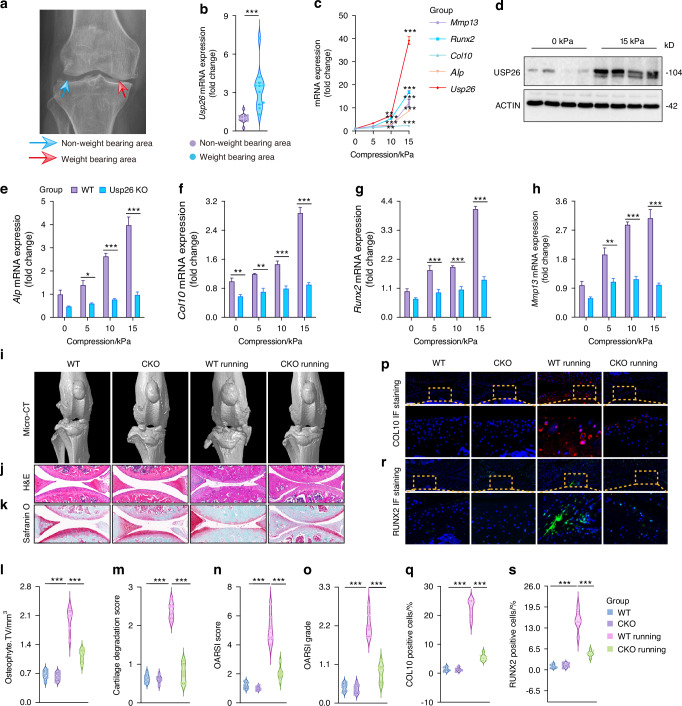
Compression loading induces USP26 to initiate chondrocyte hypertrophy and mineralization. **a** Plain radiographs of osteoarthritic knee; green arrow indicates non-weight-bearing area, while red arrow indicates weight-bearing area. **b** USP26 expression in the articular cartilages of weight-bearing and non-weight-bearing areas. **c** The mRNA expression levels of Mmp13, Runx2, Col10, Alp, and Usp26 were measured in chondrocytes following compression stimulation at levels of 0 kPa, 5 kPa, 10 kPa, and 15 kPa for a duration of 4 h. **d** The protein level of USP26 in chondrocytes subjected to 15 kPa compression stimulation for 4 h. **e**–**h** The mRNA expression of Alp, Col10, Runx2, and Mmp13 was analyzed in WT and Usp26 KO chondrocytes subjected to compression stimulation at 0 kPa, 5 kPa, 10 kPa or 15 kPa for 4 h. **i**–**k** Micro-CT, H&E staining, and Safranin O-Fast Green staining were performed on the knee joints of Usp26 CKO mice and their WT littermates, both with and without running-induced OA. Scale bar represents 300 μm. **l**–**o** The quantification of osteoarthritis development involves using scores for osteophyte formation, cartilage degradation, OARSI score, and OARSI grade. **p**–**s** Immunofluorescence analysis of COL10 and RUNX2 in the knee joints of Usp26 CKO mice and their WT littermates with or without running-induced OA. Scale bar represents 150 μm. ^*^P < 0.05, ^**^P < 0.01, ^***^P < 0.001. *P*-values were analyzed by two-tailed *t* tests in **b**, one-way ANOVA in **c**, **l**–**o**, **q**, **s** and two-way ANOVA in **e**–**h**

From the early stages of embryonic development to the manifestation of diseases in later life, mechanical loading plays a crucial role at various biological levels. It spans from macroscopic biomechanics to microscopic mechanobiology.^30^ The skeletal system, serving as the mechanical framework of the body, is continually exposed to the dynamic mechanical environment. Skeletal homeostasis is finely regulated by mechanical forces.^31^ In clinical practice, fracture healing can be impaired by insufficient mechanical loading during the postoperative fracture rehabilitation period, such as stress shielding caused by rigid fixation or limited ambulation.^22^ Additionally, prolonged mechanical loading can lead to hypertrophy and endochondral ossification of joint cartilage, which is the primary cause of osteoarthritis.^32,33^ Our study discovered that USP26 expression is elevated in the cartilage tissue of osteoarthritis, particularly in areas under compression loading (Figs. 1I and 7a, b). We also observed increased USP26 expression in the knee cartilage tissue of mice with OA induced by knee joint instability (Fig. 1l). Based on this, we hypothesize that prolonged compression loading may promote USP26 expression in chondrocytes, leading to their hypertrophic differentiation and the development of osteoarthritis.

To test this hypothesis, we stimulated primary joint articular chondrocytes with varying levels of compression loading and assessed their differentiation. The figures illustrate that, as the intensity of compressive loading increased, chondrocytes exhibited elevated expression of genes associated with hypertrophic and osteoblastic differentiation, including Runx2, Col10, Mmp13, Usp26, and Alp (Fig. 7c). This suggests that compression loading can induce chondrocyte hypertrophy and mineralization. Furthermore, whole transcriptome analysis confirmed the hypertrophic differentiation of chondrocytes induced by compression loading, as indicated by the upregulation of several genes related to hypertrophy and mineralization, such as Bmp2, Smurf1, and Smad7 (Fig. S15). GO biological process, GSEA, and KEGG pathway enrichment analysis further validated that the differentially expressed genes were mostly correlated with chondrocyte differentiation, hypertrophy, osteogenic differentiation, glucose metabolism, and mitochondrial homeostasis (Fig. S16).

Interestingly, we observed that the expression of USP26 increased in a compression loading-dependent manner along with the expression of genes associated with chondrocyte differentiation (Fig. 7c, d). To further investigate the role of USP26 in pressure-induced chondrocyte hypertrophic differentiation in vitro, we subjected WT and Usp26^−/−^ chondrocytes to compression stimulation. As expected, the knockout of Usp26 significantly inhibited the expression of compression loading-induced Alp, Col10, Runx2 and Mmp13 (Fig. 7e–h). Consistent with the finding that USP26 contributes to chondrocyte hypertrophy and mineralization by maintaining mitochondrial function (Fig. 5), we further observed that compressive loading significantly enhanced mitochondrial respiratory activity. This was demonstrated by increased basal respiration, ATP production, maximal respiratory capacity, and spare respiratory capacity (Fig. S17A–E). Moreover, the expression of key OXPHOS genes—including MT-ND1, MT-CYB, MT-CO1, and MT-ATP6—was significantly upregulated. However, Usp26 knockout markedly attenuated these effects (Fig. S17F–I).

Furthermore, to provide further evidence that long-term compression loading promotes chondrocyte hypertrophic differentiation in vivo through USP26, a running-induced OA model was used in Usp26 CKO mice and their WT littermates. This model simulates prolonged joint pressure, contributing to chondrocyte hypertrophy and the development of osteoarthritis.^34–36^ The results from micro-CT analysis, H&E staining, and Safranin O-Fast Green staining of the knee joint, along with OARIS scores, clearly indicate that knocking out Usp26 in chondrocytes significantly inhibits mechanical loading-induced OA phenotypes. This includes reductions in cartilage damage and degradation, as well as decreased osteophyte formation around the cartilage (Fig. 7i–o). Furthermore, the expression of COL10 and RUNX2 in joint tissues validates the protective effect of USP26 knockout on mechanical loading-induced OA (Fig. 7p–s). Therefore, these results demonstrated that compression loading induces USP26 to initiate chondrocyte hypertrophy and mineralization.

### Compression loading induces the expression of USP26 through phosphorylation of estrogen receptor-α at serine 118

**Fig. 8:**
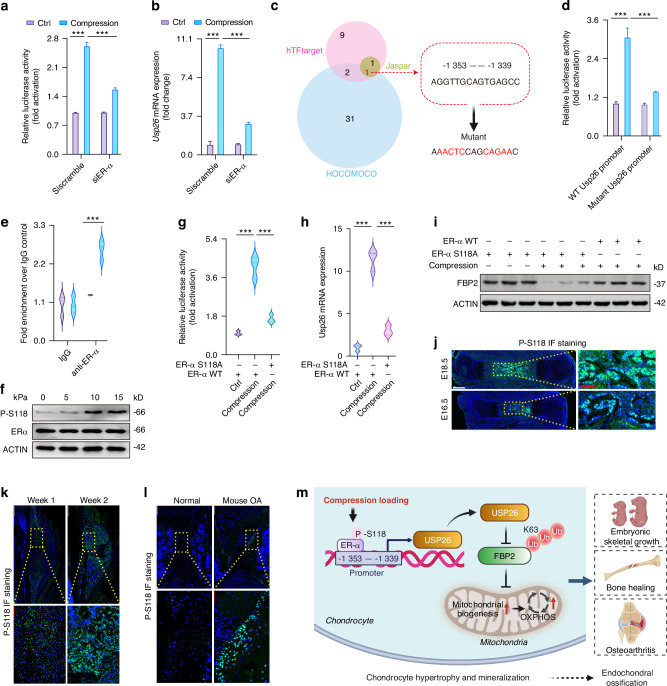
Compression loading induces the expression of USP26 through phosphorylation of estrogen receptor-α at serine 118. Dual-luciferase reporter assays to detect Usp26 promoter activity (**a**) and quantitative analysis of Usp26 mRNA expression (**b**) were conducted in chondrocytes under compression stimulation at levels of 0 and 15 kPa for a duration of 4 h, with or without ER-α knockdown. **c** The common binding sites of ER-α on the Usp26 gene promoter were further analyzed and screened through three public databases: hTFtarget, Jaspar, and HOCOMOCO. **d** The binding site mutation significantly impaired the Usp26 promoter activity induced by compression loading in primary human chondrocytes. **e** ChIP‒qPCR analysis of the binding sites of ER-α to the Usp26 gene promoter in primary human chondrocytes. **f** Western blot to detect the phosphorylation of serine 118 of ER-α under compression loading in primary human chondrocytes. The phosphorylation-deficient S118A mutation significantly attenuated the compression-induced increases in Usp26 promoter activity (**g**) and mRNA expression (**h**) in primary human chondrocyte. **i** The S118A mutation significantly restored the decreased protein level of FBP2 caused by compression loading. Immunofluorescence staining to detect phosphorylation of ER-α at S118 in chondrocytes during skeletal growth (**j**), bone fracture healing (**k**), and osteoarthritis (**l**). White and Red scale bars indicated 500 μm. **m** Schematic of USP26 facilitates endochondral ossification by driving chondrocyte hypertrophy and mineralization. ^***^P < 0.001. *P*-values were analyzed by two-way ANOVA in **a**, **b**, **d**, **e** and one-way ANOVA in **g**, **h**

Having observed that compression loading induces USP26 to initiate chondrocyte hypertrophy and mineralization, we next sought to detect the underlying mechanisms by which compression loading induces USP26 expression. Our previous study revealed that USP26 expression was decreased in osteoblast precursors collected from ovariectomized mice, and estrogen could induce USP26 in BMSCs.^12^ Additionally, estrogen receptor (ER)-α has been reported to promote chondrocyte hypertrophy and osteogenic differentiation through the GSK-3β/β-catenin pathway.^37^ Herein, we further found that ER-α knockdown significantly impaired the compression-induced upregulation of Usp26 promoter activity and mRNA expression in chondrocytes (Fig. 8a, b). Therefore, we hypothesize that ER-α is involved in the transcriptional expression of Usp26 induced by compression loading in chondrocytes.

ER-α is a transcription factor that promotes the transcriptional expression of target genes by binding to the promoter. The Genecards database has revealed that ER-α is one of the top transcription factors that bind to the Usp26 gene promoter. Furthermore, the common binding sites of ER-α on the Usp26 gene promoter were further analyzed and screened through three public databases: hTFtarget, Jaspar, and HOCOMOCO. This analysis identified the putative binding sites of ER-α on the USP26 gene promoter from −1 353 to −1 339 bp, with the base sequences of AGGTTGCAGTGAGCC (Fig. 8c). Therefore, we hypothesize that ER-α acts as a transcription factor that enhances the transcriptional expression of the Usp26 gene by binding to its promoter. To test it, the putative binding sites were mutated by site-directed mutagenesis (Fig. 8c). The results showed that the binding site mutation significantly impaired the Usp26 promoter activity induced by compression loading (Fig. 8d). To further confirm the significance of the potential binding site for compression loading-mediated transcription of Usp26, we carried out chromatin immunoprecipitation (ChIP) of the ER-α protein, and RT-qPCR to assess the enrichment of Usp26 at the putative binding sites under compression loading. The findings indicated that ER-α binds to the −1 353 to −1 339 bp regions of the Usp26 promoter, leading to an increase in Usp26 transcriptional activity (Fig. 8e).

ER-α is a transcription factor that regulates the expression of target genes in a ligand-dependent manner. Here, we demonstrate the activation of ER-α by compression loading rather than by estrogen treatment. Therefore, to further investigate the underlying mechanisms by which compression loading induces the activation of ER-α, we detected the phosphorylation of ER-α at serine 118 (S118), as S118 phosphorylation has been reported to induce ER-α activation in a ligand-independent manner.^38^ The western blot results firstly revealed that compression loading significantly induced the phosphorylation of S118 of ER-α, while having no significant impact on the total protein level of ER-α (Fig. 8f). Secondly, the phosphorylation-deficient S118A mutation^39^ significantly dampened the increased promoter activity of Usp26 induced by compression loading (Fig. 8g). Consistent with the promoter activity of Usp26, the phosphorylation-deficient S118A mutation significantly attenuated the compression-induced increase in Usp26 mRNA expression (Fig. 8h). In contrast, the S118A mutation significantly restored the decreased protein level of FBP2 caused by compression loading (Fig. 8i). Additionally, immunofluorescence staining revealed enhanced phosphorylation of ER-α at S118 in chondrocytes during skeletal growth, bone fracture healing, and osteoarthritis (Fig. 8j–l). This suggests that S118 phosphorylation of ER-α is essential for the transcriptional expression of Usp26 induced by compression loading.

## Discussion

Endochondral ossification plays a critical role in bone development, repair, and the onset of osteoarthritis.^1–4^ Chondrocyte hypertrophy and mineralization are required for endochondral ossification.^5^ Therefore, delving into the molecular mechanisms of chondrocyte hypertrophy and mineralization can enhance our comprehension of endochondral ossification and offer fresh targets for controlling skeletal development, repair, and osteoarthritis progression. This study has demonstrated that bone morphogenetic protein-USP26^12,13^ plays a role in endochondral ossification by acting as a mechanosensor and regulating chondrocyte hypertrophy and mineralization. Furthermore, our research suggests that USP26 promotes this process by increasing glucose consumption and maintaining mitochondrial biogenesis. This is achieved by reducing FBP2 levels through the inhibition of K63 ubiquitination (Fig. 8m). These findings indicate that USP26 could be a potential therapeutic target for enhancing normal skeletal growth, promoting bone fracture healing, and treating osteoarthritis.

USP26 was first identified by Wang et al. during a study on mouse spermatogonia.^40^ A human homolog with a testis-specific expression pattern has also been discovered.^41^ Several studies have found different variations in USP26 in patients with nonobstructive azoospermia or severe oligozoospermia, suggesting that changes in USP26 may contribute to male infertility, although there is some controversy surrounding this claim.^42–44^ Further research has revealed that USP26 also plays a role in other organs, such as promoting esophageal squamous cell carcinoma metastasis by stabilizing Snail.^45^ Additionally, USP26 has been identified as a key regulator of bone homeostasis, coordinating bone formation and resorption.^12,13^ It is able to stabilize β-catenin, thus promoting the osteogenic activity of mesenchymal cells (MSCs). Knocking out USP26 in MSCs significantly inhibits the expression of bone differentiation-related genes such as runx2 and ALP.^12,13^ Chondrocytes, which are a type of intermediate cells derived from mesenchymal stem cells in the bone formation process. This study has further demonstrated that USP26 can participate in endochondral ossification by regulating chondrocyte hypertrophy and mineralization. These findings broaden the functional role of USP26 in regulating skeletal system homeostasis and the occurrence of bone diseases.

Endochondral ossification is an energy-dependent anabolic process.^24^ The proliferation and differentiation of chondrocytes and the formation of extracellular matrix require a large amount of energy. Therefore, stable energy metabolism in chondrocytes is key to maintaining the normal process of chondrocyte hypertrophic differentiation and mineralization.^24^ Glycolysis and oxidative phosphorylation are the two main energy production pathways in eukaryotic organisms. Although glycolysis contributes to over 60% of the energy in chondrocytes, glucose oxidative phosphorylation is still necessary to maintain normal proliferation, differentiation, and collagen synthesis in chondrocytes.^24^ Comprehensive transcriptomic analysis revealed that glucose metabolism-related pathways, including gluconeogenesis, glycolysis, and oxidative phosphorylation, were significantly enriched in the differentially expressed mRNAs between WT and Usp26^−/−^ chondrocytes during both physiological endochondral ossification and differentiation induction. Subsequent cellular experiments demonstrated that the knockout of Usp26 significantly reduced glucose intake and ATP production in chondrocytes. These findings indicate that the absence of USP26 indeed hinders the uptake and utilization of glucose by chondrocytes. The process of converting glucose into ATP entails several steps: initially, glucose within the cytoplasm undergoes glycolysis to produce pyruvate. Under anaerobic conditions, pyruvate is converted into lactate, generating a small amount of ATP. Conversely, under aerobic conditions, pyruvate enters the mitochondria and undergoes the citric acid cycle, electron transport chain, ultimately resulting in complete oxidation into water and carbon dioxide, and the production of a large amount of ATP. This study revealed that the knockout of USP26 not only led to a decrease in mitochondrial quantity but also caused damage to the mitochondrial network and impaired mitochondrial biogenesis. In addition, analysis of the extracellular acidification rate (ECAR) showed that Usp26 knockout markedly reduced basal glycolysis, maximal glycolytic capacity, and glycolytic reserve in chondrocytes (Fig. S18). The ratio of OCR to ECAR under basal metabolic conditions represents the cell’s preference for oxidative phosphorylation over aerobic glycolysis when mitochondrial oxygen consumption is coupled to energy production.^46^ We found little difference in the OCR/ECAR ratio between WT and Usp26 knockout chondrocytes at the basal state (Fig. S18), suggesting that USP26 deletion causes a simultaneous reduction in both glycolysis and oxidative phosphorylation in chondrocytes.

USP26, as a deubiquitinating enzyme, regulates protein expression primarily through deubiquitination modification, thereby affecting protein stability.^12^ This study observed that the knockout of USP26 significantly increased the expression of FBP2 in chondrocytes. FBP2 is a well-known key enzyme involved in gluconeogenesis. Cytosolic FBP2 counteracts the elevated glycolysis associated with the “Warburg effect”, while nuclear-localized FBP2 inhibits mitochondrial biogenesis and respiration, independent of its catalytic activity, by suppressing the expression of TFAM.^28^ FBP2 deficiency in avian or mice muscle cells promotes glucose uptake, upregulates glycolysis, enhances mitochondrial respiration, and increases the number of mitochondria.^47,48^ On the other hand, augmentation of FBP2 results in destabilization of mitochondrial network dynamics.^49^ In line with this, herein we found that TFAM expression was significantly decreased in USP26 KO chondrocytes. Furthermore, inhibition of FBP2 overexpression significantly rescues glucose uptake, ATP generation, lactate release, and mitochondrial biogenesis in USP26 KO chondrocytes. Additionally, inhibiting FBP2 enhances the endochondral ossification process of articular cartilage in osteoarthritis. However, Further research is required to investigate whether inhibiting FBP2 can rescue endochondral ossification in USP26^−/−^ mice during embryonic skeletal growth and bone fracture healing, as well as to study the changes in glucose metabolism processes in vivo.

In further investigating the molecular mechanisms of USP26 expression in chondrocytes, this study found that USP26 acts as a mechanosensing factor, contributing to chondrocyte hypertrophy and endochondral ossification induced by compression loading. The skeletal system, as the weight-bearing organ of the body, undergoes physiological endochondral ossification due to compression loading during growth and development.^50^ The repair of fractures recapitulates the bone growth and development process, with endochondral ossification of cartilage being a principal mechanism of repair. Therefore, insufficient compression loading can impair fracture healing.^22^ Additionally, the primary cause of OA is long-term abnormal compression loading on articular cartilage, which promotes chondrocyte hypertrophy and endochondral ossification.^2,51^ It is crucial to identify factors that make chondrocytes sensitive to mechanical loading to understand the effects of mechanical signals on biology and find targets for regulating bone development, bone healing, and OA. This study found a significant increase in USP26 expression in knee joint cartilage tissue of OA patients and in an experimental animal model of OA. Furthermore, USP26 was primarily located in mechanically loaded areas of knee joint cartilage tissue and played a crucial role in promoting chondrocyte hypertrophy and endochondral ossification induced by long-term repetitive mechanical loading. While Usp26 CKO mice showed significant dwarfism during the embryonic period and impaired bone fracture healing compared to their wild-type (WT) littermates, further validation is required to determine the direct role of USP26 in promoting endochondral ossification through mechanical loading during skeletal development and bone healing. Additionally, while we have found that S118 phosphorylation of ER-α is crucial for the transcriptional expression of Usp26 induced by compression loading, these findings were derived solely from in vitro experiments. It is still unclear whether S118 phosphorylation of ER-α is necessary for USP26 expression in response to mechanical loading in vivo. Moreover, additional research is required to ascertain whether S118 phosphorylation of ER-α plays a role in the chondrocyte hypertrophic differentiation and mineralization during endochondral ossification, despite previous reports indicating that ER-α promotes this process.^37^

In conclusion, this study has discovered that USP26 participates in endochondral ossification by driving chondrocyte hypertrophy and mineralization, thereby stimulating skeletal development, bone fracture healing and the occurrence of osteoarthritis. Mechanistically, USP26 acts as a mechanosensor that supports chondrocyte hypertrophy and mineralization by regulating glucose consumption through the reduction of FBP2. These findings suggest that targeting USP26 could hold promise as a therapeutic strategy to enhance healthy skeletal growth, facilitate bone fracture healing, and manage osteoarthritis.

## Materials and methods

### Human normal and OA cartilage sample collection

Normal human cartilage samples were gathered from 18 patients between the ages of 51 and 78 who had tibial plateau fractures but did not show any signs of osteoarthritis (OA). The mean age of these patients was 64 ± 12.5 years, with a total of 10 patients involved in the study. Osteoarthritic cartilage samples, on the other hand, were collected from 21 patients between the ages of 55 and 79 who underwent TKA surgery for end-stage OA of the knees. These patients had Kellgren-Lawrence grade 4, joint destruction, and obliterated joint space. The mean age of these patients was 67 ± 11.6 years, also with a total of 10 patients involved in the study. After collection, the cartilage samples were immediately transported to the laboratory in a sterile container filled with phosphate-buffered saline to maintain their viability and prevent any degradation. At the laboratory, the samples were carefully dissected to remove any remaining connective tissue, fat, or blood. The cleaned samples were then stored at -80°C for further analysis.

The research procedure was approved by the Shanghai Ruijin Hospital review board for human knee cartilage samples, and all patients included in the study signed an informed consent prior to enrollment, and ethics approval was granted by the Ruijin Hospital Ethics Committee, Shanghai Jiao Tong University School of Medicine, with the project number of 1.0/2022-11-02. Any knees that had previous bone fractures or knee surgeries, including knee replacement procedures, ligamentoplasty, and cartilage repair procedures, were excluded from the study.

### Generation of Usp26 conditional knock-out mice

Usp26 floxed mice were provided by GemPharmatech Co., Ltd (Nanjing, China). The mice were constructed as follows: Firstly, two sgRNAs were constructed and transcribed in vitro to target the introns on both sides of the floxed region of Usp26. In addition, a donor vector with the loxp fragment was designed and constructed in vitro. Following this, Cas9 mRNA, sgRNA, and the donor were coinjected into zygotes. The zygotes were then transferred into the oviduct of pseudopregnant ICR females at 0.5 dpc. F0 mice were born after 19–21 days of transplantation. All the offspring of ICR females (F0 mice) were identified by PCR and sequencing of tail DNA, and positive F0 mice were crossed with C57BL/6J mice to produce heterozygous mice.

GemPharmatech Co., Ltd (Nanjing, China) provided Col2α1-CreERT2 mice, which contained a mutated estrogen receptor fused to Cre that could be activated by tamoxifen. Usp26 floxed mice were crossed with Col2α1-CreERT2 mice for at least three generations to generate inducible chondrocyte-specific Usp26-deficient mice (Col2α1-CreERT2; Usp26^f/f^). The Usp26-floxed littermates were used as controls (WT). Genotypes were determined by PCR amplification of purified tail genomic DNA. Animal experiments were conducted following the approved protocol by the LENSCI Biotechnology (Kunshan) Co., LTD Animal Care and Use Committee and in strict compliance with the Ministry of Science and Technology of the People’s Republic of China’s Animal Care guidelines [IACUC protocol number: 20231223-0085-01].

### Skeletal staining

Skeletal preparation and staining were carried out following a previous report.^12^ Usp26 was deleted in Col2α1-CreERT2; Usp26 f/f embryos through a single injection of tamoxifen (Sigma-Aldrich, T5648) at a dosage of 0.125 mg/g of body weight^52^ into pregnant mothers at E11.5. At E16.5, Usp26 CKO mice (derived from tamoxifen-injected Col2α1-CreERT2; Usp26 f/f embryos) and their littermate controls (from Col2α1-CreERT2; Usp26 f/f embryos without tamoxifen injection) were dissected., with the removal of the skin, and the resulting samples were then placed in acetone for 48 h after being fixed overnight in 95% ethanol. After 3 days, the samples were washed with water and stained for 2 days using a solution of 0.005% ARS and 0.015% Alcian blue dissolved in ethanol. After rinsing with water, the samples were immersed in a mixture of 20% glycerol and 1% KOH until the skeletons became clearly visible. For long-term storage, the samples were gradually transferred to 50%, 80%, and finally 100% glycerol.

### Histology and histochemical staining

The bones were fixed in 4% paraformaldehyde overnight at 4 °C for H&E and Safranin O-Fast green histological staining. They were then decalcified in 0.25 mol/L EDTA at pH 7.4 for 2–4 weeks and embedded in paraffin. Osteoarchitectural analysis was performed by staining paraffin sections with H&E. Specifically, the sections were stained with hematoxylin for 5 min and eosin for 30 s. To stain acid mucopolysaccharides and collagen fibers in the bone matrix, Safranin O-Fast green was used.

### Immunohistochemical and immunofluorescence staining

Paraffin-embedded cartilage tissues were utilized for the immunohistochemical analysis of USP26 expression. In short, paraffin sections were treated to remove wax, rehydrated, and incubated with antigen retrieval solution. After that, they were washed in tris buffered saline (TBS). Endogenous peroxidase activity was inhibited by immersing the sections in a mixture of methanol and 3% hydrogen peroxide (v/v) for 10 min. Unspecific antibody binding was blocked by incubating the sections in TBS supplemented with 2% bovine serum albumin (w/v) for half an hour. Following this, the sections were incubated overnight with a primary antibody against USP26. The slides were then exposed to horseradish peroxidase (HRP)-conjugated streptavidin for 30 min, and antibody binding was visualized based on HRP activity using the colored substrate diaminobenzidine (DAB Kit; Invitrogen, Carlsbad, CA). Finally, the slides were counterstained with hematoxylin.

To assess the expression of USP26, COL10, MMP13, VEGF, and RUNX2 through immunofluorescence staining, paraffin sections underwent dewaxing, rehydration, and permeabilization using 0.5% Triton X-100. This was followed by the blocking of non-specific antibodies. The bone tissue was then incubated with specific antibodies against USP26, MMP13, VEGF, COL10, and RUNX2 at 4 °C overnight. Next, the sections were incubated with secondary antibodies at room temperature for 30 min. The secondary antibodies used were specific antibodies for hamster, sheep, and rabbit. The paraffin sections were labeled with DAPI to stain the nuclei. Finally, the sections were examined using laser scanning confocal microscopy.

### Von Kossa staining and Masson staining

The E16.5 Usp26 CKO mice and their littermate controls underwent Von Kossa staining on undecalcified sections, following previously reported methods.^53^ In this procedure, sections were exposed to 5% AgNO_3_ for 1 h, resulting in silver deposits where calcium was present. The staining was then fixed using 5% Na_2_S_2_O_3_ and counterstained with haematoxylin, causing the mineralized bone area to appear black. For Masson staining,^54^ thin sections were prepared and dewaxed, followed by staining with Weigert iron hematoxylin staining solution (HT1079, Sigma) for 5–10 min. The cells were differentiated using an acidic ethanol differentiation solution (1% hydrochloric acid in alcohol, Sigma) for 5–15 s and washed with water. Next, the samples were exposed to a 1% lithium carbonate aqueous solution (G1841, Solarbio) for 3–5 min and washed with distilled water for 1 min. Ponceau acid fuchsin staining buffer (LZ-12686, Guidechem) was applied for 5–10 min, followed by washing with a weak acid working solution (0.1%–0.3% acetic acid solution, Sigma) for 1 min. The samples were then washed with a phosphomolybdic acid solution (20 wt% in ethanol, 319 279, Sigma) for 1–2 min and again with the weak acid working solution for 1 min. Subsequently, the sections were exposed to an aniline blue staining solution (2.5% in 2% acetic acid, B8563, Sigma) for 1–2 min, followed by another wash with the weakly acidic working solution for 1 min. After dehydration, the sheets were sealed using a neutral resin.

### Real-time PCR analysis

Trizol reagent (Invitrogen, Carlsbad, CA, USA) was used for the extraction of total RNA. For reverse transcription of mRNA, 1 μg of total RNA was used with the Prime-Script RT reagent kit (#RR036A, Takara Biotechnology, Japan). Quantitative real-time PCR was conducted to amplify the cDNA using the SYBR Premix Ex Tag kit (Takara Biotechnology, Japan) and ABI 7500 Sequencing Detection System (Applied Biosystems, Foster City, CA, USA). β-actin was utilized as the endogenous control to quantify mRNA expression. The primer sequences for real-time PCR are presented in Table S1.

### ChIP-PCR

The ChIP procedure was performed as previously described utilizing a ChIP assay kit (Millipore Sigma, Burlington, MA). With 10 μL of ER-α antibody (CST, #13258) or negative control anti-IgG (CST, #2729), fragmented chromatin (200 μg) was incubated overnight to form immune complexes, which were coupled to Protein A beads. The DNA-star Lasergene 15.2 core suite (DNA-star, Madison, WI) was used to design primers for the putative ER-α-binding regions −1 353 to −1 339 bp on the Usp26 promoter. All primers were designed through Primer-BLAST, and primer sequences are included in Table S2. Immunoprecipitated DNA was analyzed through RT‒qPCR as previously described. With the ΔΔCt method and normalization to % input, fold changes were calculated and are presented as fold enrichment relative to the control Rabbit IgG.

### Protein isolation and western blot analysis

Protein isolation and western blot analysis was carried out according to previously reported methods.^55^ After the femur tissues were dissected from the mice, the periosteal surface was removed from the femurs using tweezers. The femur tissues were then washed three times in PBS and ground using FastPrep-24™ 5G (MP Biochemicals). The ground tissues were then resuspended in RIPA buffer containing protease inhibitor. After being lysed in RIPA buffer for 20 min on ice, the lysates were collected and centrifuged at 14 000 × *g* for 15 min. The supernatants, which contained the proteins, were collected for western blot analysis. Each sample, containing 10 μg of total protein, was separated by SDS-PAGE in a 10% gel and transferred onto PVDF membranes (EMD Millipore Corporation, US). After blocking with 5% nonfat dry milk in Tris-buffered saline with 1‰ Tween (TBST), the membranes were incubated overnight at 4 °C with primary antibodies against USP26. After three washes with TBST, the membrane was incubated with horseradish peroxidase-conjugated secondary antibodies (Jackson). The antibody-antigen complexes were then visualized using Immobilon reagents (Millipore).

### The whole transcriptome analysis

#### RNA isolation and library preparation

The bone tissues were minced and homogenized using the FastPrep-24™ 5G (MP Biochemicals) machine. After homogenization, the tissues were suspended in TRIreagent (Sigma-Aldrich, T9424). Following the manufacturer’s protocol, RNA was isolated. The NanoDrop 2000 spectrophotometer (Thermo Scientific, USA) was used to assess the purity and quantity of the RNA. The Agilent 2100 Bioanalyzer (Agilent Technologies, Santa Clara, CA, USA) was utilized to evaluate the integrity of the RNA. The VAHTS Universal V6 RNA-seq Library Prep Kit was used to construct libraries, following the manufacturer’s instructions. OE Biotech Co., Ltd (Shanghai, China) performed the transcriptome sequencing and analysis.

#### RNA sequencing and differentially expressed genes analysis

RNA sequencing and analysis of differentially expressed genes were conducted in the following manner. The Illumina Novaseq 6000 platform was used to sequence the libraries, resulting in 150 bp paired-end reads. Each sample produced ~6G raw reads. The fastp tool was used to remove low-quality reads and obtain clean reads in fastq40 format. Around 4G clean reads were obtained for each sample. These clean reads were then mapped to the house mouse genome using HISAT2. FPKM values were calculated for each gene, and HTSeq-count was used to obtain the read counts for each gene. R (v 3.2.0) was utilized to perform PCA analysis in order to assess the biological duplication of the samples.

For the differential expression analysis, DESeq243 was used. Significantly differentially expressed genes (DEGs) were determined based on a *P* value of < 0.05 and a fold change of >1.5 or <0.66. Hierarchical cluster analysis of the DEGs was carried out in R (v 3.2.0) to illustrate the expression patterns of genes in different groups and samples. To visualize the expression of upregulated or down-regulated DEGs, a radar map of the top 30 genes was generated using the R package ggradar.

Enrichment analysis of DEGs was performed for GO, KEGG pathway, Reactome, and WikiPathways using R (v 3.2.0) and the hypergeometric distribution. Significant enriched terms were selected and visualized through column diagrams, chord diagrams, and bubble diagrams using R (v 3.2.0).

#### Cell culture

Chondrocytes were extracted from the knee articular cartilage of neonatal mice using collagenase digestion, as described in previous studies.^56^ The articular cartilage tissues were sliced into small pieces measuring 1 mm^3^, then treated with 0.25% trypsin for 0.5 h, followed by 0.2% type II collagenase for 4 h in a shaking incubator with a shaking speed of 90 r/min. The released chondrocytes were collected and cultured in Dulbecco’s modified Eagle’s medium (DMEM)/F12 media supplemented with 10% fetal bovine serum (FBS) and 1% penicillin-streptomycin solution at 37 °C in a humidified incubator with 5% CO_2_. To maintain the characteristics of the cartilage, chondrocytes within two passages were used.

To induce chondrocyte differentiation, the cells were cultured with a supplement of insulin, transferrin, and sodium selenite (ITS) for a period of 3 weeks. During this time, the culture medium was changed every 3 days. After the 3-week period, the cells were exposed to DMEM/10% FBS containing 4 mmol/L inorganic phosphate (Pi) for 2 days. In order to inhibit mitochondrial function, either FCCP (MCE Cat. #HY-100410, 1 μmol/L) or Oligomycin A (MCE Cat. #HY-16589, 2 μmol/L) was added to the culture medium.

#### Mouse OA model and intra-articular injection treatment

Tamoxifen (Sigma; 100 μg per gram of body weight) was administered to both 8-week-old Col2a1-CreERT;Usp26^fl/fl^ mice and their Usp26^fl/fl^ littermates via intraperitoneal injection for a period of 5 days. Subsequently, surgical procedure was conducted to establish an experimental OA model, following a previously documented protocol.^23^ Eight weeks after the surgery, the subjects were evaluated using the OARSI system^57^ to assess the severity of OA. The assessment was performed by a single observer who was blinded to the experimental groups.

One week after the surgery, the Usp26 CKO (Col2a1-CreERT;Usp26^fl/fl^) mice were subjected to intra-articular injections once 4 week. Each injection consisted of 20 μL of Fbp2 shRNA or shRNA-Control, depending on the respective experimental group. After 8 weeks, we analyzed the knee joints of the mice.

#### Micro-CT analysis

A high-resolution SkyScan1172 Micro-CT machine (Bruker, Belgium) was used to conduct CT scans of mouse knee joints with the parameters set to 100 kVp, 100 Ma, and 10.0 μm/pixel. Conebeam reconstruction software (NRecon; Bruker) was employed to reconstruct sequential tomographs from raw data, which were then used to calculate parameters for trabecular bone in the subchondral bone. All measurements were carried out in accordance with the guidelines of the American Society for Bone and Mineral Research.

#### Flexcell compression

Flexcell compression was carried out in accordance with a previous study.^58^ Matrigel matrix (Corning Incorporated, Corning, NY) was used to seed primary mouse chondrocytes (6 × 10^5^ cells/well) in BioPress compression plates (Flexcell International, Burlington, NC) at 37 °C, with DMEM/F12 medium supplemented with 10% FBS, 1% penicillin-streptomycin. Following a 48-h incubation in a cell culture incubator, the chondrocytes were subjected to cyclical compression using pulses of 5, 10 and 15 kPa for 4 h at a frequency of 0.1 Hz using the FlexCell FX-5000. As a control, cells were maintained without compression (0 kPa).

#### Forced running to induce mouse knee OA

Mice in the running mice groups were subjected to electronic stimulation in order to make them run on a treadmill (Melquest, Toyama, Japan), following the protocol mentioned in a previous publication.^34^ The mice were compelled to run at a speed of 25 m/min for 30 min per day, 5 days a week, for a continuous period of 8 weeks. The mice in the non-running THM and C57BL/6 group, on the other hand, stayed in their cage.

#### Fbp2 knockdown with short hairpin RNA (shRNA)

To silence Fbp2, shRNA specifically targeting mouse Fbp2 and a negative control shRNA were ligated into the lentiviral vector pLV[shRNA]-EGFP:T2A:Puro-U6 separately using restriction enzyme digestion and ligation.

The validity of all vectors was assessed using Sanger Sequencing and Restriction Enzyme Digestion Assay during the final stage of vector construction. To produce lentivirus, the silencing vector was co-transfected with pLV/helpr-SL3 (gag/pol element), pLV/helper-SL4 (pRev element), and pLV/helper-SL5 (pVSVG element) in HEK293T cells using the calcium phosphate transfection method.

After 48 h, the supernatant containing the lentiviral particles was collected and concentrated for purification, resulting in the final lentivirus for transduction. The titers were determined using the Lenti-X p24 Rapid Titer Kit. The efficiency of silencing was assessed using either western blot or RT-qPCR.

#### MitoTracker staining

WT and Usp26 knockout chondrocytes were incubated with 100 nmol/L MitoTracker Green FM probe (Thermo Fisher Scientific, M7514) at 37 °C for 30 min. After the staining process was finished, the cells were delicately washed three times with PBS. The cells were then detached from the plates and a single cell suspension was ensured. The samples were analyzed using a laser scanning confocal microscope (ZEISS).

#### ATP measurements

ATP production was measured using the ATP Determination Kit (Thermo Fisher Scientific, A22066) following the manufacturer’s protocol. Briefly, cells were homogenized in lysis buffer consisting of 1% Triton X-100, 0.1% SDS, 150 mmol/L NaCl, and 50 mmol/L Tris-HCl at pH 7.5. The lysis buffer was supplemented with a protease and phosphatase inhibitor cocktail (Thermo Fisher Scientific, 78445). Data were obtained through luminescence using a Promega Glomax 20/20 luminometer. Multiple replicate wells were used for each experiment, and the data were normalized to protein concentration.

#### Citrate synthase activity

The activity of Citrate synthase was measured on total cell extracts using the Citrate Synthase Activity Assay Kit (Abcam, ab119692). In summary, 100 μL of cell lysates were added to the microplate strips, which were pre-coated, sealed, and then incubated at room temperature for 3 h. The wells were then aspirated and washed twice with wash buffer. The activity solution was added to the wells, and the plate was immediately read every 20 s for 30 min at a wavelength of 412 nm using a plate reader (TECAN Infinite m200 PRO).

#### Oxygen consumption rate (OCR) plate assay

WT and Usp26^–/–^ chondrocytes were isolated and seeded in 96-well plates (black transparent plate) at a density of 5 × 10^4^ per well. The cells were cultured overnight in a 37 °C, 5% CO_2_ incubator. The medium was removed (taking care not to peel the cells), and 100 µL of medium was added to Blank 1, Blank 2, and Blank 3, along with 100 µL of Working solution. The plate was then placed into a fluorescent enzyme-labeled instrument preset at 37 °C and cultured for 30 min. After preheating the medium to 37 °C, 10 µL of medium was added to Blank 1, Blank 2, and Blank 3. Next, 10 µL of the experimental drug diluted with medium was added to the samples. Immediately after adding the experimental drug, 1 drop of Mineral Oil was added to each well. The plate was then placed into a fluorescent enzyme-labeled instrument at 37 °C and cultured for 5 min. The enzyme marker was read continuously every 10 min for 200 min using the following parameters: Ex: 500 nm, Em: 650 nm, bottom reading mode. The fluorescence intensity was recorded to calculate the OCR value.

#### Glucose uptake assay

150 μL of WT and Usp26^–⁄–^ chondrocytes (1.0 × 10^5^ cells/mL) were seeded and cultured in 96-well plates overnight in MEM medium containing 10% FBS. The plates were incubated at 37 °C in a 5% CO_2_ incubator. Afterward, the supernatant was removed, and the wells were rinsed twice with 150 μL of enzyme-free, serum-free MEM medium. Each well was then filled with 150 μL of enzyme-free, serum-free MEM medium and incubated at 37 °C in a 5% CO_2_ incubator for 15 min. The supernatant was again removed, and 150 μL of enzyme-free, serum-free MEM medium along with MEM high-glucose, serum-free medium were added to the respective wells, along with the Probe solution. The plates were kept in a 5% CO_2_ incubator at 37 °C for 15 min. Once again, the supernatant was removed, and the wells were cleaned with 150 μL of 1× WI Solution at 4 °C. This cleaning process was repeated three times. The TECAN Infinite m200 PRO instrument was used for testing, with an excitation wavelength of 488 nm and an emission wavelength of 520 nm.

#### Lactate secretion assay

Lactate secretion was determined using the TECAN Infinite m200 PRO. Briefly, WT and Usp26^–⁄–^ chondrocytes were seeded in 6 cm plates with 6 replicates each. The next day, the medium was removed and the cells were washed with PBS three times. Then, three 15-mL centrifuge tubes were prepared, each containing 20 μL 2dH_2_O, 3 mmol/L standards, and samples, as well as 1 mL enzyme working solution and 200 μL TMB solution. The mixture was thoroughly mixed and incubated for 10 min at 37 °C. Afterwards, 2 mL stopping solution was added and the OD 530 nm was measured using the TECAN Infinite m200 PRO. The lactate content in the cells was calculated using the following formula: Lactate secretion (nmol/prot) = (Ameasurement – ABLANK)/(Astandard - ABLANK) * Cstandard/Cpr (the concentration of protein samples).

#### mtDNA content

Total DNA was extracted from cell samples using Wizard Genomic DNA Purification Kit (Promega, A1120) according to the manufacturer’s instructions. To quantify mtDNA copy number, real-time PCR was performed using a on a ViiA7 Real-Time.

PCR system from Applied Biosystems against MT-ND1 (forward primer, 5′- CGAGACCGGTTCGATTTTGC-3′, and reverse primer, 5′- ATCTCCCAAGCGAAGATGCC-3′) as the standard for mtDNA. The β-globin (HBB) was used as the nuclear gene (nDNA) normalizer for calculation of the mtDNA/nDNA ratio. A fragment of HBB gene was amplified using forward primer, 5′- GCTGAGGAGAAGGCAGCTATC-3′, and reverse primer, 5′- TCTAATCCGGGGGTTTCCCA-3′. The relative mtDNA content was calculated using the formula: mtDNA content = 1/2^ΔCt^, where ΔCt= Ct_mtDNA_−Ct_HBB_.

### Statistical analysis

All data representative of three independent experiments are present as mean ± S.E.M. We used two-tailed *t*-tests to determine significances between two groups. We did analyses of multiple groups by one- or two-way ANOVA with Bonferroni post-test of GraphPad prism version 5. For all statistical tests, we considered *P*-value < 0.05 to be statistically significant.

## Supplementary information

Supplementary materials

## Data Availability

All data needed to evaluate the conclusions in the paper are present in the paper and/or the Supplementary materials related to this paper may be requested from the authors.

## References

[1] Kronenberg, H. M. Developmental regulation of the growth plate. *Nature* **423**, 332–336 (2003). 12748651 10.1038/nature01657

[2] Kawaguchi, H. Endochondral ossification signals in cartilage degradation during osteoarthritis progression in experimental mouse models. *Mol. Cells* **25,** 1–6 (2008). 18319608

[3] Saito, T. et al. Transcriptional regulation of endochondral ossification by HIF-2alpha during skeletal growth and osteoarthritis development. *Nat. Med.* **16,** 678–686 (2010). 20495570 10.1038/nm.2146

[4] Hosaka, Y. et al. Notch signaling in chondrocytes modulates endochondral ossification and osteoarthritis development. *Proc. Natl. Acad. Sci. USA* **110,** 1875–1880, (2013). 23319657 10.1073/pnas.1207458110 PMC3562777

[5] Morita, K. et al. Reactive oxygen species induce chondrocyte hypertrophy in endochondral ossification. *J. Exp. Med.* **204,** 1613–1623 (2007). 17576777 10.1084/jem.20062525 PMC2118643

[6] Stickens, D. et al. Altered endochondral bone development in matrix metalloproteinase 13-deficient mice. *Development* **131,** 5883–5895 (2004). 15539485 10.1242/dev.01461 PMC2771178

[7] Zelzer, E. et al. VEGFA is necessary for chondrocyte survival during bone development. *Development* **131**, 2161–2171 (2004). 15073147 10.1242/dev.01053

[8] Wang, X. et al. Inhibition of overactive TGF-beta attenuates progression of heterotopic ossification in mice. *Nat. Commun.* **9**, 551 (2018). 29416028 10.1038/s41467-018-02988-5 PMC5803194

[9] Yoshida, M. et al. The transcription factor Foxc1 is necessary for Ihh-Gli2-regulated endochondral ossification. *Nat. Commun.* **6**, 6653 (2015). 25808752 10.1038/ncomms7653

[10] Lanske, B. et al. PTH/PTHrP receptor in early development and Indian hedgehog-regulated bone growth. *Science* **273**, 663–666 (1996). 8662561 10.1126/science.273.5275.663

[11] Mak, K. K., Chen, M. H., Day, T. F., Chuang, P. T. & Yang, Y. Wnt/beta-catenin signaling interacts differentially with Ihh signaling in controlling endochondral bone and synovial joint formation. *Development* **133**, 3695–3707 (2006). 16936073 10.1242/dev.02546

[12] Li, C. et al. The osteoprotective role of USP26 in coordinating bone formation and resorption. *Cell Death Differ.* **29**, 1123–1136 (2022). 35091692 10.1038/s41418-021-00904-x PMC9177963

[13] Xu, Y. et al. USP26 combats age-related declines in self-renewal and multipotent differentiation of BMSC by maintaining mitochondrial homeostasis. *Adv. Sci.* **11**, e2406428 (2024). 10.1002/advs.202406428 PMC11600297 39377219

[14] Tang, J. et al. Activating the osteoblastic USP26 pathway alleviates multi-organ fibrosis by decreasing insulin resistance. *Adv. Sci.* **13**, e12424 (2025). 10.1002/advs.202512424 PMC12915124 41417635

[15] Sun, J. et al. Histone demethylase LSD1 is critical for endochondral ossification during bone fracture healing. *Sci. Adv.* **6**, eaaz1410 (2020). 33148658 10.1126/sciadv.aaz1410 PMC7673679

[16] Gibson, G. Active role of chondrocyte apoptosis in endochondral ossification. *Microsc. Res. Tech.* **43**, 191–204 (1998). 9823004 10.1002/(SICI)1097-0029(19981015)43:2<191::AID-JEMT10>3.0.CO;2-T

[17] Atkins, G. J. et al. Sclerostin is a locally acting regulator of late-osteoblast/preosteocyte differentiation and regulates mineralization through a MEPE-ASARM-dependent mechanism. *J. Bone Miner. Res.* **26**, 1425–1436 (2011). 21312267 10.1002/jbmr.345 PMC3358926

[18] Peng, Z. et al. MiR-20a: a mechanosensitive microRNA that regulates fluid shear stress-mediated osteogenic differentiation via the BMP2 signaling pathway by targeting BAMBI and SMAD6. *Ann. Transl. Med.* **10**, 683 (2022). 35845505 10.21037/atm-22-2753 PMC9279817

[19] Zhuang, X. et al. Differential effects on lung and bone metastasis of breast cancer by Wnt signalling inhibitor DKK1. *Nat. Cell Biol.* **19**, 1274–1285 (2017). 28892080 10.1038/ncb3613

[20] Hendrickx, G. et al. Piezo1 inactivation in chondrocytes impairs trabecular bone formation. *J. Bone Miner. Res.* **36**, 369–384 (2021). 33180356 10.1002/jbmr.4198

[21] Qiu, M. et al. Black phosphorus accelerates bone regeneration based on immunoregulation. *Adv. Sci.* **11**, e2304824 (2024). 10.1002/advs.202304824 PMC10767454 37953457

[22] Jia, S. et al. Insufficient mechanical loading downregulates Piezo1 in chondrocytes and impairs fracture healing through ApoE-induced senescence. *Adv. Sci.* **11**, e2400502 (2024). 10.1002/advs.202400502 PMC11633519 39418070

[23] Kamekura, S. et al. Osteoarthritis development in novel experimental mouse models induced by knee joint instability. *Osteoarthr. Cartil.* **13**, 632–641 (2005). 10.1016/j.joca.2005.03.004 15896985

[24] Stegen, S. et al. HIF-1alpha metabolically controls collagen synthesis and modification in chondrocytes. *Nature* **565**, 511–515 (2019). 30651640 10.1038/s41586-019-0874-3 PMC7195049

[25] Sherratt, H. S. Mitochondria: structure and function. *Rev. Neurol.* **147**, 417–430 (1991). 1962047

[26] Li, X. et al. Ultrasensitive sensors reveal the spatiotemporal landscape of lactate metabolism in physiology and disease. *Cell Metab.* **35**, 200–211.e209 (2023). 36309010 10.1016/j.cmet.2022.10.002 PMC10560847

[27] Xue, K. et al. The mitochondrial calcium uniporter engages UCP1 to form a thermoporter that promotes thermogenesis. *Cell Metab.* **34**, 1325–1341.e1326 (2022). 35977541 10.1016/j.cmet.2022.07.011

[28] Huangyang, P. et al. Fructose-1,6-bisphosphatase 2 inhibits sarcoma progression by restraining mitochondrial biogenesis. *Cell Metab.* **31**, 174–188e177 (2020). 31761563 10.1016/j.cmet.2019.10.012 PMC6949384

[29] Pluska, L. et al. The UBA domain of conjugating enzyme Ubc1/Ube2K facilitates assembly of K48/K63-branched ubiquitin chains. *EMBO J.* **40**, e106094 (2021). 33576509 10.15252/embj.2020106094 PMC7957398

[30] Iskratsch, T., Wolfenson, H. & Sheetz, M. P. Appreciating force and shape-the rise of mechanotransduction in cell biology. *Nat. Rev. Mol. Cell Biol.* **15**, 825–833 (2014). 25355507 10.1038/nrm3903 PMC9339222

[31] Wang, L. et al. Mechanical sensing protein PIEZO1 regulates bone homeostasis via osteoblast-osteoclast crosstalk. *Nat. Commun.* **11**, 282 (2020). 31941964 10.1038/s41467-019-14146-6 PMC6962448

[32] Li, C. et al. Double-stranded RNA released from damaged articular chondrocytes promotes cartilage degeneration via Toll-like receptor 3-interleukin-33 pathway. *Cell Death Dis.* **8**, e3165 (2017). 29095435 10.1038/cddis.2017.534 PMC5775407

[33] Zhang, H. et al. Mechanical overloading promotes chondrocyte senescence and osteoarthritis development through downregulating FBXW7. *Ann. Rheum. Dis.* **81**, 676–686 (2022). 35058228 10.1136/annrheumdis-2021-221513

[34] Yamagishi, K. et al. Activation of the renin-angiotensin system in mice aggravates mechanical loading-induced knee osteoarthritis. *Eur. J. Histochem.* **62**, 2930 (2018). 30043596 10.4081/ejh.2018.2930 PMC6060485

[35] Lee, Y. J. et al. Evaluation of osteoarthritis induced by treadmill-running exercise using the modified Mankin and the new OARSI assessment system. *Rheumatol. Int.* **31**, 1571–1576 (2011). 20490805 10.1007/s00296-010-1520-4

[36] Siebelt, M. et al. Hsp90 inhibition protects against biomechanically induced osteoarthritis in rats. *Arthritis Rheum.* **65**, 2102–2112 (2013). 23666904 10.1002/art.38000

[37] Yan, X. et al. ERα/β/DMP1 axis promotes trans-differentiation of chondrocytes to bone cells through GSK-3β/β-catenin pathway. *J. Anat.* **240**, 1152–1161 (2022). 35081258 10.1111/joa.13612 PMC9119614

[38] Kato, S. et al. Activation of the estrogen receptor through phosphorylation by mitogen-activated protein kinase. *Science* **270**, 1491–1494 (1995). 7491495 10.1126/science.270.5241.1491

[39] Du, Z. et al. The sequence-structure-function relationship of intrinsic ERalpha disorder. *Nature* **638**, 1130–1138 (2025). 39779860 10.1038/s41586-024-08400-1 PMC11864982

[40] Wang, P. J., McCarrey, J. R., Yang, F. & Page, D. C. An abundance of X-linked genes expressed in spermatogonia. *Nat. Genet.* **27**, 422–426 (2001). 11279525 10.1038/86927

[41] Wosnitzer, M. S. et al. Ubiquitin Specific Protease 26 (USP26) expression analysis in human testicular and extragonadal tissues indicates diverse action of USP26 in cell differentiation and tumorigenesis. *PLoS One* **9**, e98638 (2014). 24922532 10.1371/journal.pone.0098638 PMC4055479

[42] Stouffs, K., Lissens, W., Tournaye, H., Van Steirteghem, A. & Liebaers, I. Possible role of USP26 in patients with severely impaired spermatogenesis. *Eur. J. Hum. Genet.* **13**, 336–340 (2005). 15562280 10.1038/sj.ejhg.5201335

[43] Tian, H. et al. Disruption of ubiquitin specific protease 26 gene causes male subfertility associated with spermatogenesis defects in mice†. *Biol. Reprod.* **100**, 1118–1128 (2019). 30561524 10.1093/biolre/ioy258 PMC6698735

[44] Sakai, K. et al. Usp26 mutation in mice leads to defective spermatogenesis depending on genetic background. *Sci. Rep.* **9**, 13757 (2019). 31551464 10.1038/s41598-019-50318-6 PMC6760205

[45] Li, L., Zhou, H., Zhu, R. & Liu, Z. USP26 promotes esophageal squamous cell carcinoma metastasis through stabilizing Snail. *Cancer Lett.* **448**, 52–60 (2019). 30763716 10.1016/j.canlet.2019.02.007

[46] Wei, Y. et al. Aerobic glycolysis is the predominant means of glucose metabolism in neuronal somata, which protects against oxidative damage. *Nat. Neurosci.* **26**, 2081–2089 (2023). 37996529 10.1038/s41593-023-01476-4

[47] Osipova, E. et al. Loss of a gluconeogenic muscle enzyme contributed to adaptive metabolic traits in hummingbirds. *Science* **379**, 185–190 (2023). 36634192 10.1126/science.abn7050

[48] Park, H. J. et al. The essential role of fructose-1,6-bisphosphatase 2 enzyme in thermal homeostasis upon cold stress. *Exp. Mol. Med.* **52**, 485–496 (2020). 32203098 10.1038/s12276-020-0402-4 PMC7156669

[49] Pietras, L., Stefanik, E., Rakus, D. & Gizak, A. FBP2-a new player in regulation of motility of mitochondria and stability of microtubules in cardiomyocytes. *Cells* **11**, 1170 (2022). 35626746 10.3390/cells11101710 PMC9139521

[50] Skedros, J. G., Hunt, K. J., Hughes, P. E. & Winet, H. Ontogenetic and regional morphologic variations in the turkey ulna diaphysis: implications for functional adaptation of cortical bone. *Anat. Rec. A Discov. Mol. Cell Evol. Biol.* **273**, 609–629 (2003). 12808646 10.1002/ar.a.10073

[51] Talts, J. F. et al. Endochondral ossification is dependent on the mechanical properties of cartilage tissue and on intracellular signals in chondrocytes. *Ann. N. Y. Acad. Sci.* **857**, 74–85 (1998). 9917833 10.1111/j.1749-6632.1998.tb10108.x

[52] Moon, E. H. et al. TMEM100 is a key factor for specification of lymphatic endothelial progenitors. *Angiogenesis* **23**, 339–355 (2020). 32112176 10.1007/s10456-020-09713-1

[53] Chen, K. et al. Osteocytic HIF-1alpha pathway manipulates bone micro-structure and remodeling via regulating osteocyte terminal differentiation. *Front. Cell Dev. Biol.* **9**, 721561 (2021). 35118061 10.3389/fcell.2021.721561 PMC8804240

[54] Qiu, M. et al. 3D biomimetic calcified cartilaginous callus that induces type H vessels formation and osteoclastogenesis. *Adv. Sci.* **10**, e2207089 (2023). 10.1002/advs.202207089 PMC10238192 36999832

[55] Zhao, S. et al. Desferrioxamine alleviates UHMWPE particle-induced osteoclastic osteolysis by inhibiting caspase-1-dependent pyroptosis in osteocytes. *J. Biol. Eng.* **16**, 34 (2022). 36482442 10.1186/s13036-022-00314-8 PMC9733322

[56] Gosset, M., Berenbaum, F., Thirion, S. & Jacques, C. Primary culture and phenotyping of murine chondrocytes. *Nat. Protoc.* **3**, 1253–1260 (2008). 18714293 10.1038/nprot.2008.95

[57] Pritzker, K. P. et al. Osteoarthritis cartilage histopathology: grading and staging. *Osteoarthr. Cartil.* **14**, 13–29 (2006). 10.1016/j.joca.2005.07.014 16242352

[58] Deng, R. et al. Periosteal CD68(+) F4/80(+) macrophages are mechanosensitive for cortical bone formation by secretion and activation of TGF-beta1. *Adv. Sci.* **9**, e2103343 (2022). 10.1002/advs.202103343 PMC8787385 34854257

