# Supplementary material for: Ubiquitin-specific protease 26 facilitates endochondral ossification by driving chondrocyte hypertrophy and mineralization
Source: Bone Res. 2026 Apr 9;14:41. doi: 10.1038/s41413-026-00517-5 (PMC13065777; doi:10.1038/s41413-026-00517-5)
Supplement: Supplementary file 1 — Supplementary materials [file 41413_2026_517_MOESM1_ESM.pdf]

Supplementary Materials for

Ubiquitin-specific protease 26 facilitates endochondral ossification by driving chondrocyte hypertrophy and mineralization

Changwei Li *et al.*

*** Corresponding authors:**

Email: lcw11876@rjh.com.cn (C. Li); 17712485172@yzu.edu.cn (G. Tang); lfdeng@shsmu.edu.cn (L. Deng)

This PDF file includes:

Figures S1 to S18

Tables S1 to S2

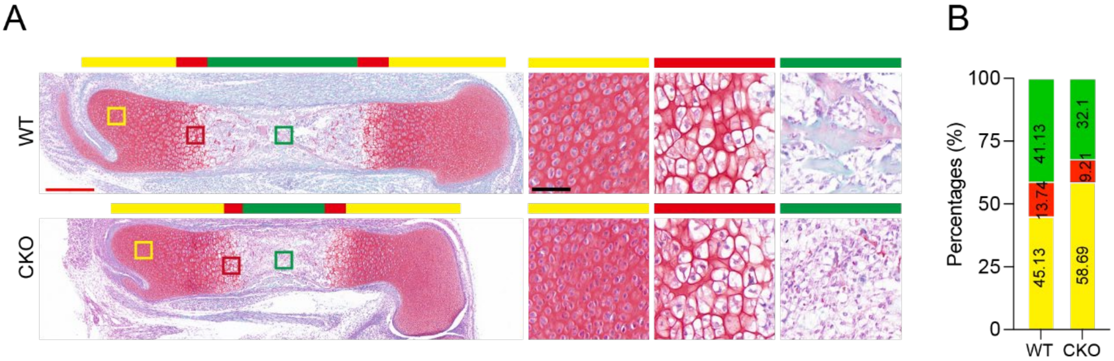

Figure S1. (**A**) Safranin O-Fast Green staining was performed on whole femurs from Usp26 CKO and WT littermates at E18.5. Inset boxes highlight regions in the three panels on the right, representing the proliferative zone, hypertrophic zone, and bone

area, indicated by the yellow, red, and green bars, respectively. The red and black scale bars represent 300 μm and 50 μm, respectively. (**B**) The percentage of the proliferative zone (yellow), hypertrophic zone (red), and bone area (green) relative to the total femur length was measured in Usp26 CKO and WT littermate embryos.

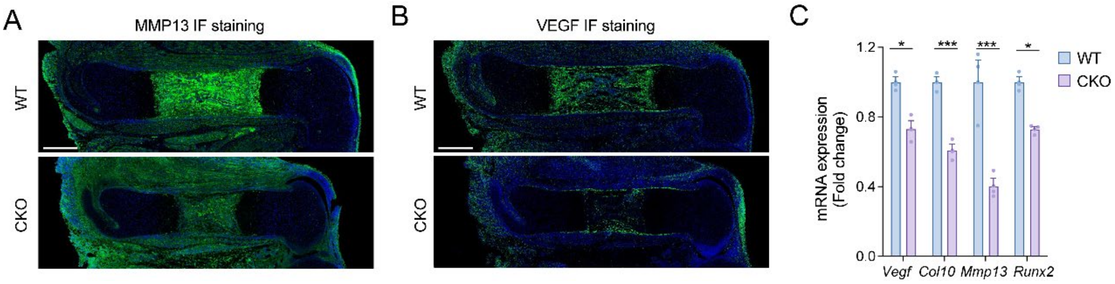

Figure S2. (A&B) Immunofluorescence staining was performed on MMP13 and VEGF on full femurs of Usp26 CKO and WT littermate embryos. Scale bars represent 300 μm. (C) The mRNA expression of Vegf, Col10, Mmp13, and Runx2 in the femurs of Usp26 CKO and WT littermate embryos were analyzed. **P<0.05*, ****P<0.001*. *P*-values were analyzed by two-tailed *t* tests.

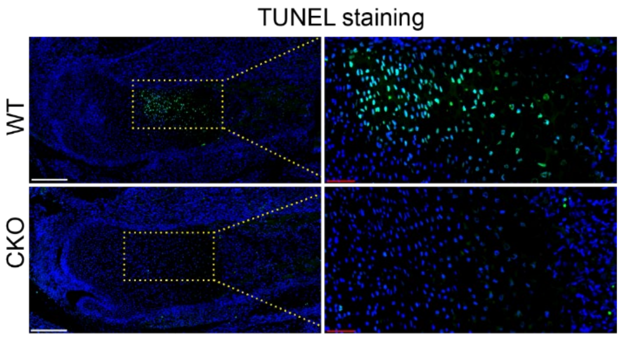

Figure S3. TUNEL staining was performed on femur sections from Usp26 CKO

and WT littermates at E18.5. White and red scale bars indicate 500 μm and 50 μm, respectively.

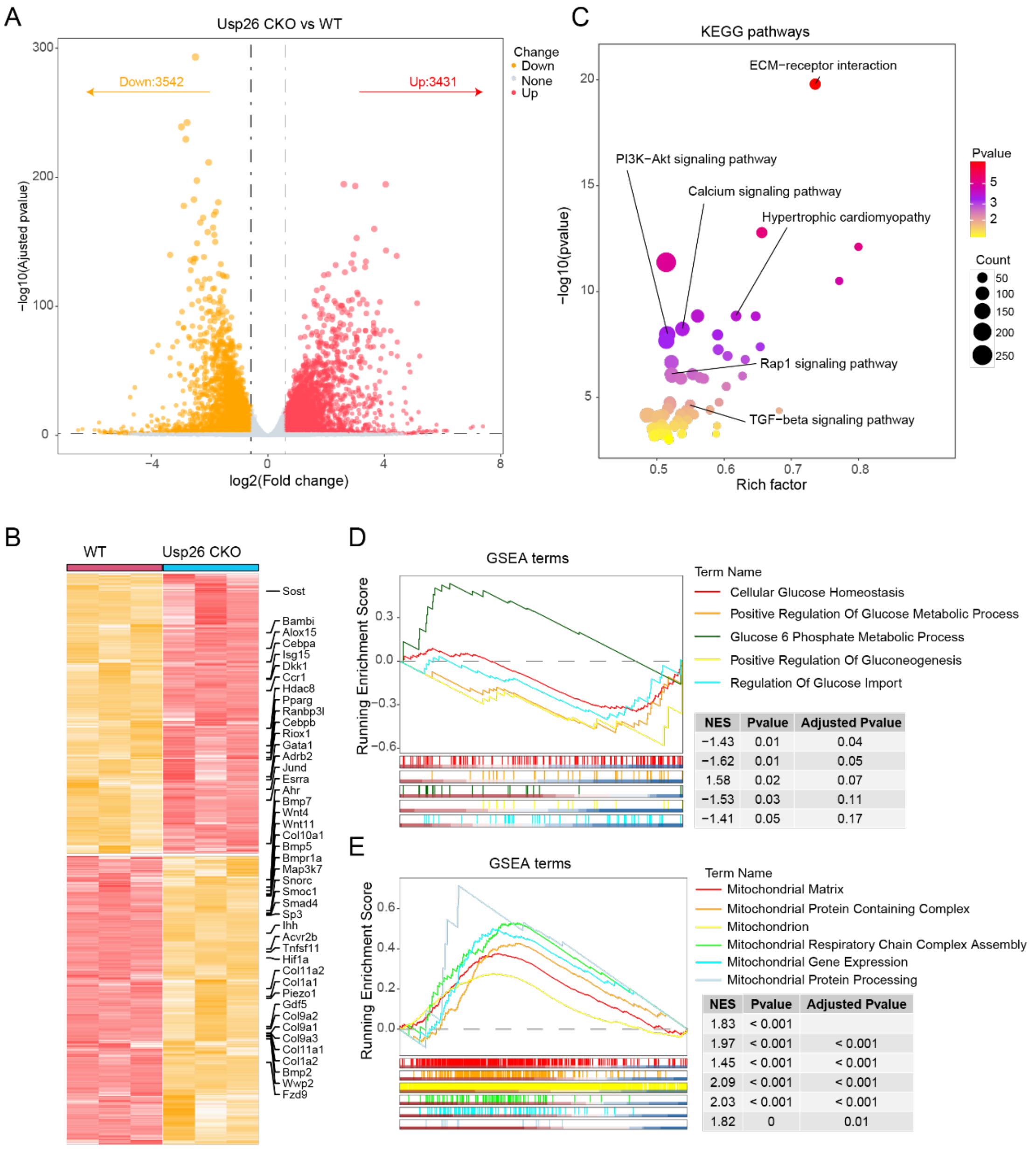

Figure S4. Transcriptome analysis was conducted on femurs obtained from Usp26 CKO and WT littermates at E18.5. (**A**) The volcano plot indicates that the deletion of Usp26 in chondrocytes resulted in the downregulation of 3542 genes and the upregulation of 3431 genes in Usp26 CKO femurs compared to WT littermate controls.

(**B**) Heatmap of gene expression. (**C**) KEGG pathway enrichment analysis was utilized to identify differentially expressed genes. (**D&E**) GSEA confirms a significant correlation between USP26 and key processes in glucose metabolism (D), and mitochondrial biogenesis (E).

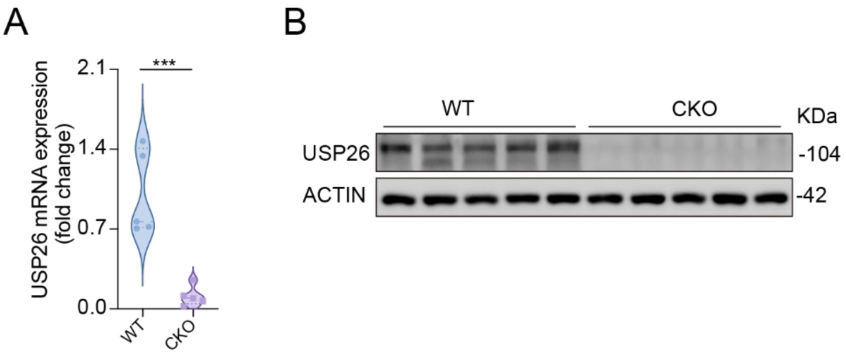

Figure S5. (A&B) USP26 gene and protein expression in cartilage calluses from Usp26 CKO mice and their WT littermates were evaluated using real-time PCR (A) and western blot analysis (B), respectively. ****P<0.001*. *P*-values were analyzed by two-tailed *t* tests.

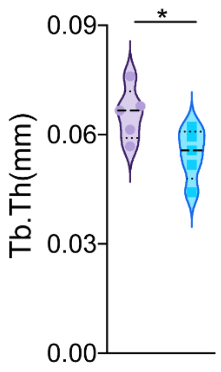

Figure S6. Quantitative analysis was conducted on the trabecular thickness (Tb. Th) of

femur callus from Usp26 CKO mice and their WT littermates at 2 weeks post-fracture. *$P<0.05$*. *P*-values were analyzed by two-tailed *t* tests.

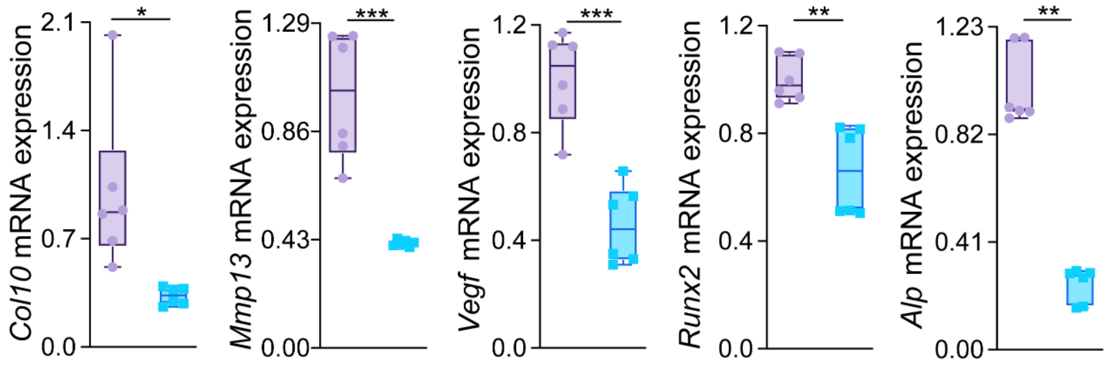

Figure S7. The mRNA expression of Col10, Mmp13, Vegf, Runx2, and Alp in femur callus from Usp26 CKO mice and their wild-type littermates at 2 weeks post-fracture. *$P<0.05$*, **$P<0.01$*, ***$P<0.001$*. *P*-values were analyzed by two-tailed *t* tests.

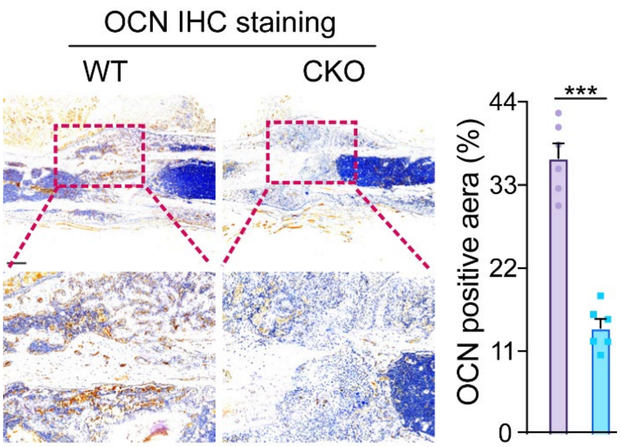

Figure S8. Immunohistochemical staining of OCN in femur callus from Usp26 CKO mice and their wild-type littermates at 2 weeks post-fracture. Scale bars represent 500 µm. ***$P<0.001$*. *P*-values were analyzed by two-tailed *t* tests.

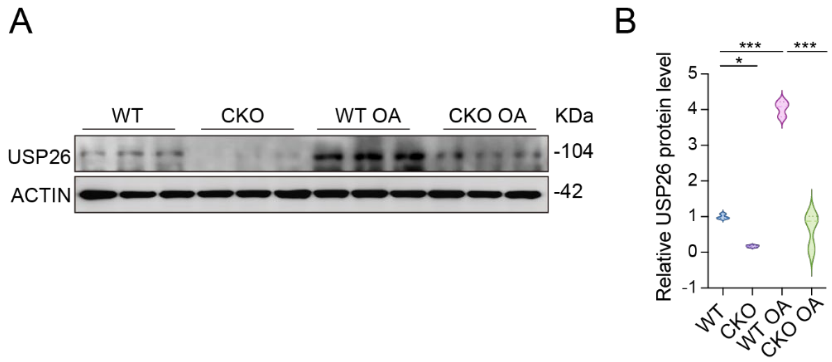

Figure S9. Western blot analysis was performed to detect USP26 expression in the joint cartilage of Usp26 CKO mice and their WT littermates, with or without surgical induction of OA. *$P<0.05$, ***$P<0.001$. *P*-values were analyzed by one-way ANOVA.

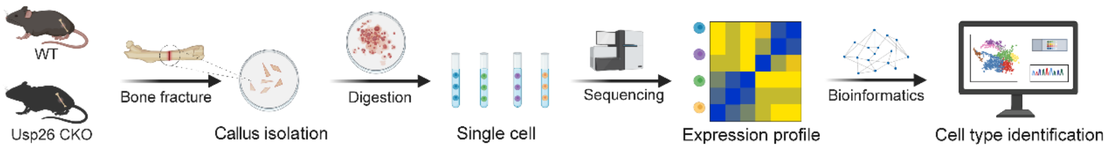

Figure S10. A schematic illustrating the scRNA-seq workflow was created using callus tissues harvested one week after fracture.

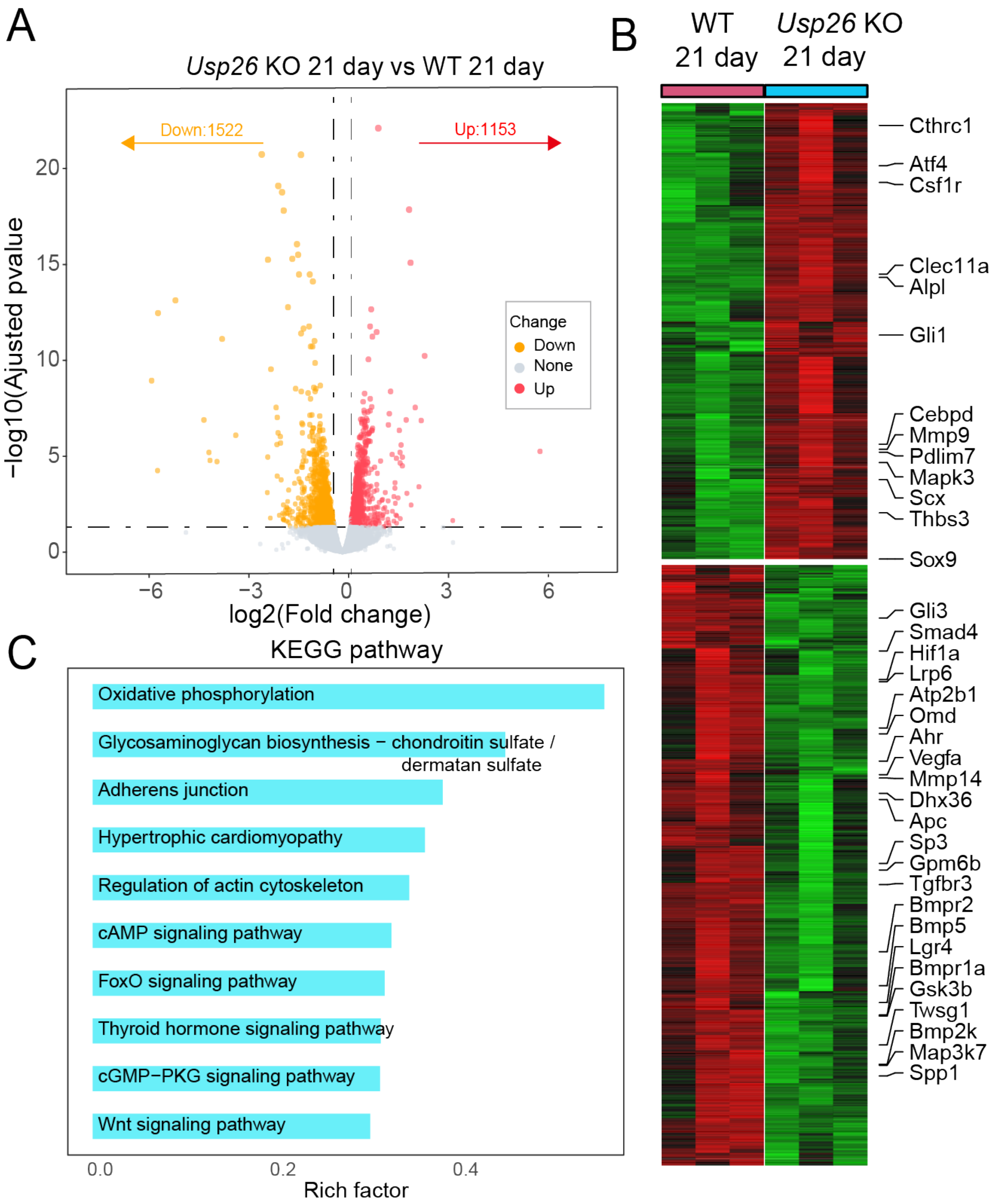

Figure S11. USP26 facilitates endochondral ossification in cultured chondrocytes. (**A&B**) The volcano plot and heatmap revealed differential gene expression in WT and Usp26 KO chondrocytes following 21 days of differentiation. (**C**) KEGG pathway enrichment analysis was utilized to identify differentially expressed genes.

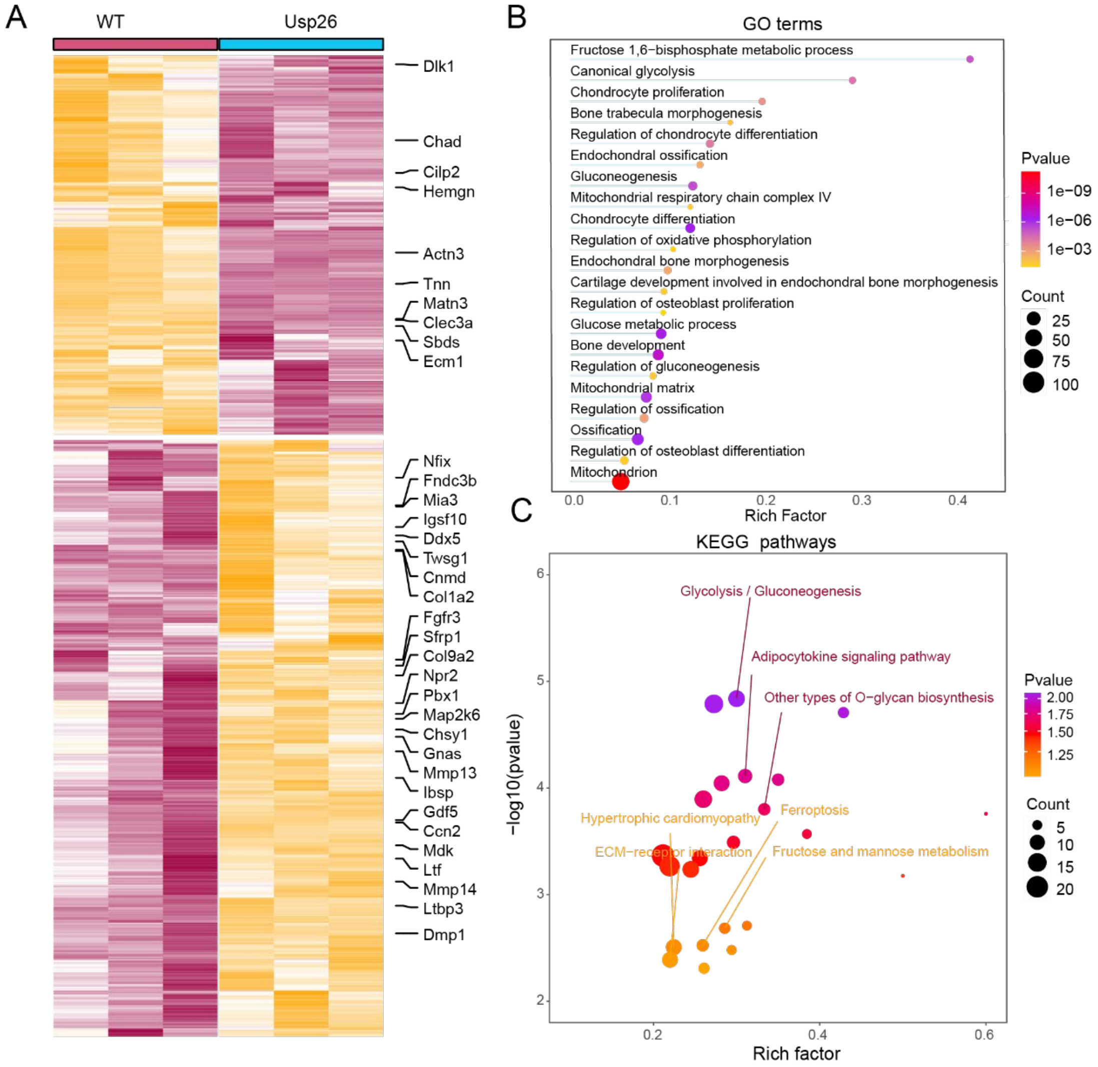

Figure S12. Proteomic sequencing was performed on the femur of both WT and USP26 CKO mice at E16.5. (**A**) 290 were upregulated and 457 were downregulated. Some of the differentially expressed proteins, such as Mmp13, Dmp1, and Col1, were found to be associated with processes like chondrocyte hypertrophy and osteogenesis. (**B**) Gene Ontology (GO) analysis confirmed that the deficiency of USP26 affects chondrocyte differentiation, endochondral ossification, and processes related to glucose metabolism, including gluconeogenesis. The fructose 1,6−bisphosphate metabolic process exhibited the highest enrichment factor. (**C**) Kyoto Encyclopedia of Genes and

Genomes (KEGG) enrichment analysis also indicated the involvement of fructose and mannose metabolism.

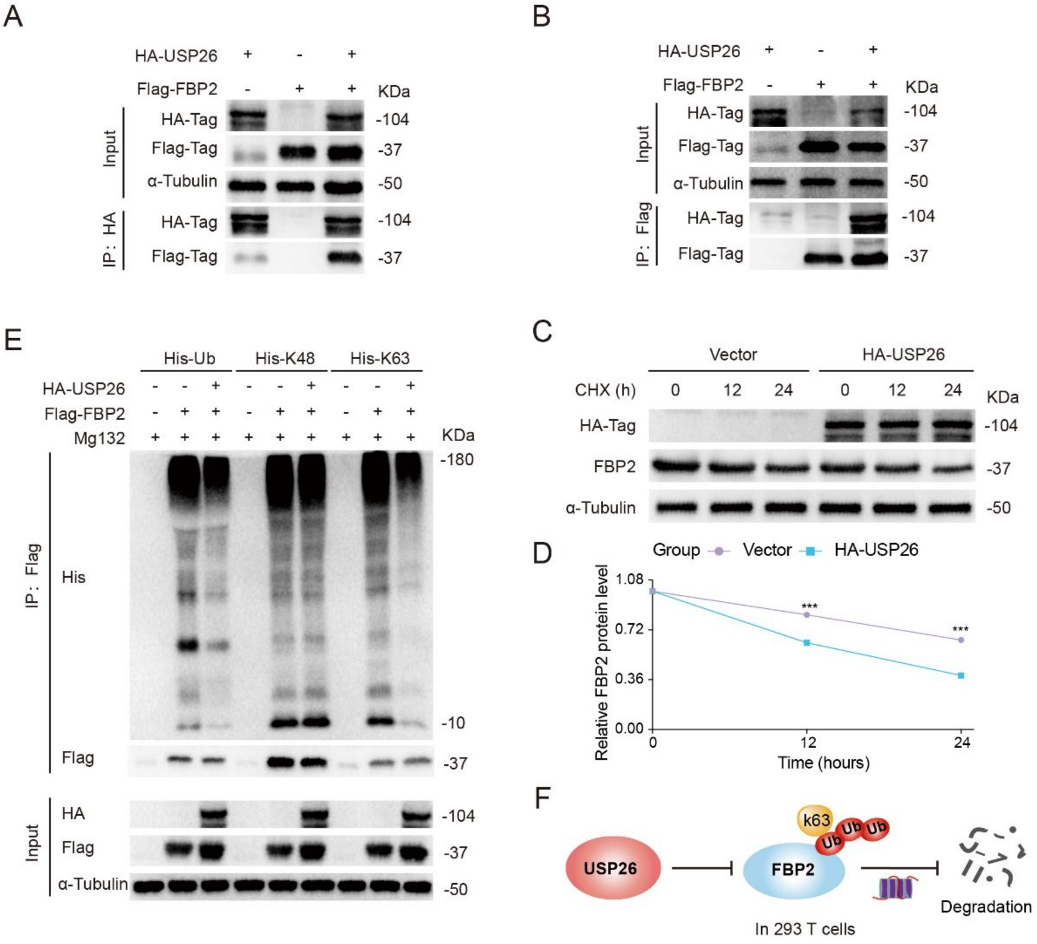

Figure S13. USP26 inhibits K63 ubiquitination-mediated stabilization of FBP2 in 293 T cells. (**A&B**) Co-IP of HA-USP26 with ectopically expressed Flag-tagged FBP2 in 293 T cells. (**C**) Western blot analysis of the protein level of FBP2 in 293 T cells with or without USP26 overexpression in the presence of cycloheximide (CHX) for indicated time intervals. (**D&E**) USP26 inhibits FBP2 to undergo K63 (Lys63)-linked ubiquitination. (**F**) The schematic graph indicates that USP26 inhibits FBP2 to undergo

K63 (Lys63)-linked ubiquitination and stabilization in 293 T cells. ****P<0.001*. *P*-values were analyzed by two-way ANOVA.

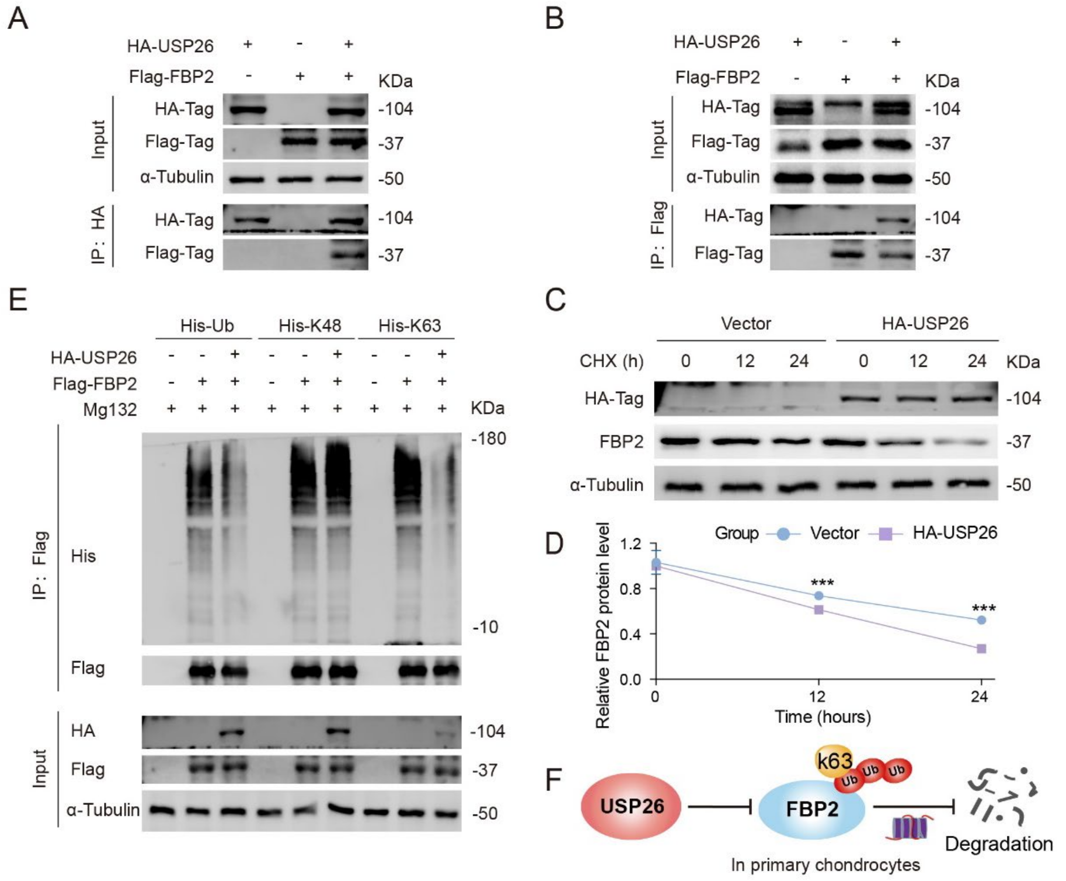

Figure S14. USP26 inhibits K63 ubiquitination-mediated stabilization of FBP2 in chondrocytes. (**A&B**) Co-IP of HA-USP26 with ectopically expressed Flag-tagged FBP2 in chondrocytes. (**C**) Western blot analysis of the protein level of FBP2 in chondrocytes with or without USP26 overexpression in the presence of cycloheximide (CHX) for indicated time intervals. (**D&E**) USP26 inhibits FBP2 to undergo K63 (Lys63)-linked ubiquitination. (**F**) The schematic graph indicates that USP26 inhibits FBP2 to undergo K63 (Lys63)-linked ubiquitination and stabilization in primary

chondrocytes. ***$P<0.001$. P-values were analyzed by two-way ANOVA.

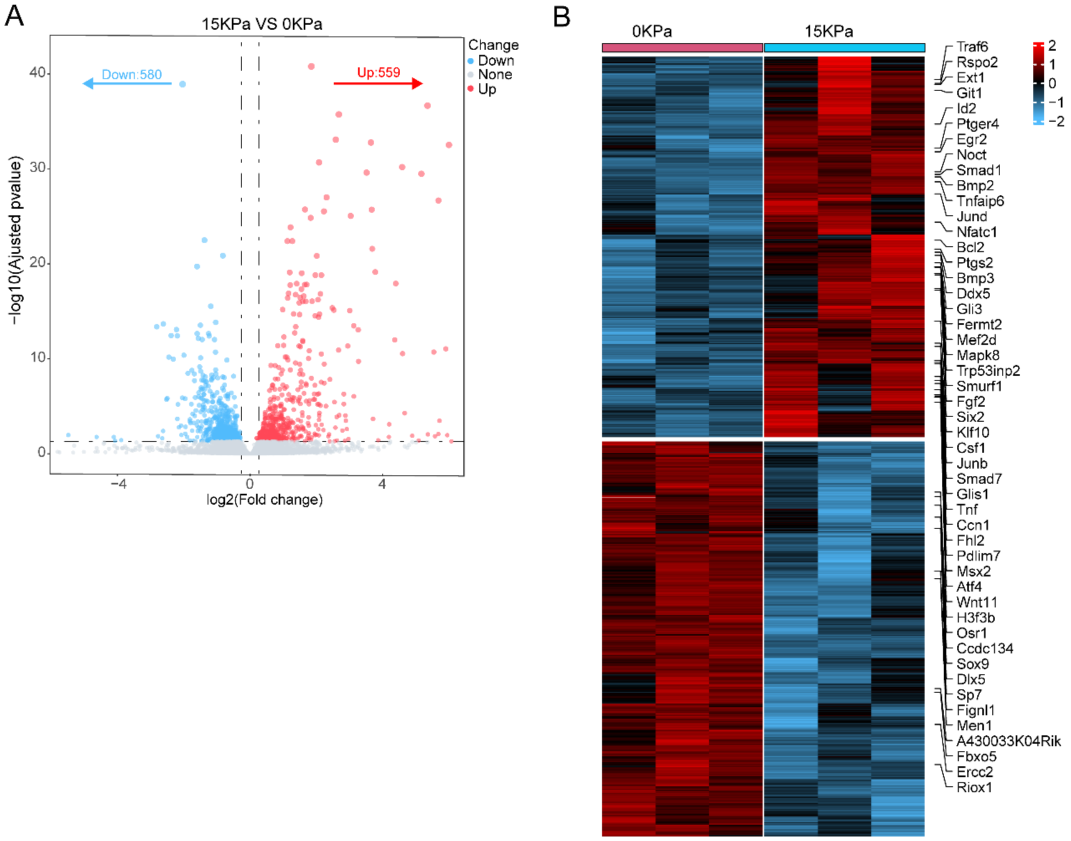

S15. (**A&B**) The volcano plot and heatmap revealed differential gene expression in WT and Usp26 KO chondrocytes following compression stimulation.

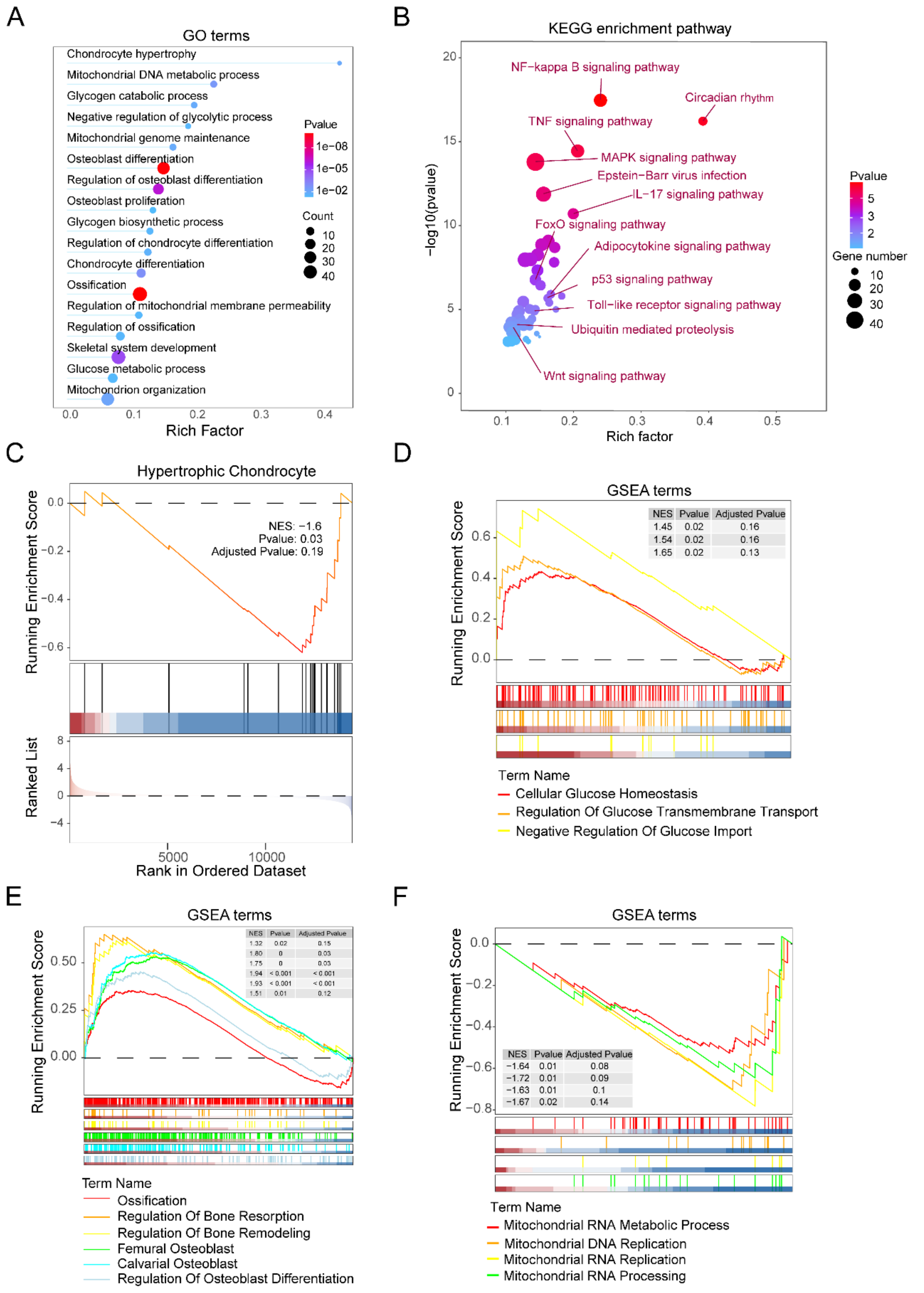

S16. (**A**) GO biological process analysis was used to identify the differentially expressed genes in chondrocytes between 0 KPa and 15 KPa stimulation. (**B**) KEGG pathway enrichment analysis was utilized to identify differentially expressed genes.

(**C&E**) GSEA confirms a significant correlation between USP26 and key processes in chondrocyte hypertrophy and ossification in the presence of compression stimulation. (**D&F**) GSEA confirms a significant correlation between USP26 and key processes in glucose metabolism (D), and mitochondrial biogenesis (F) following compression stimulation.

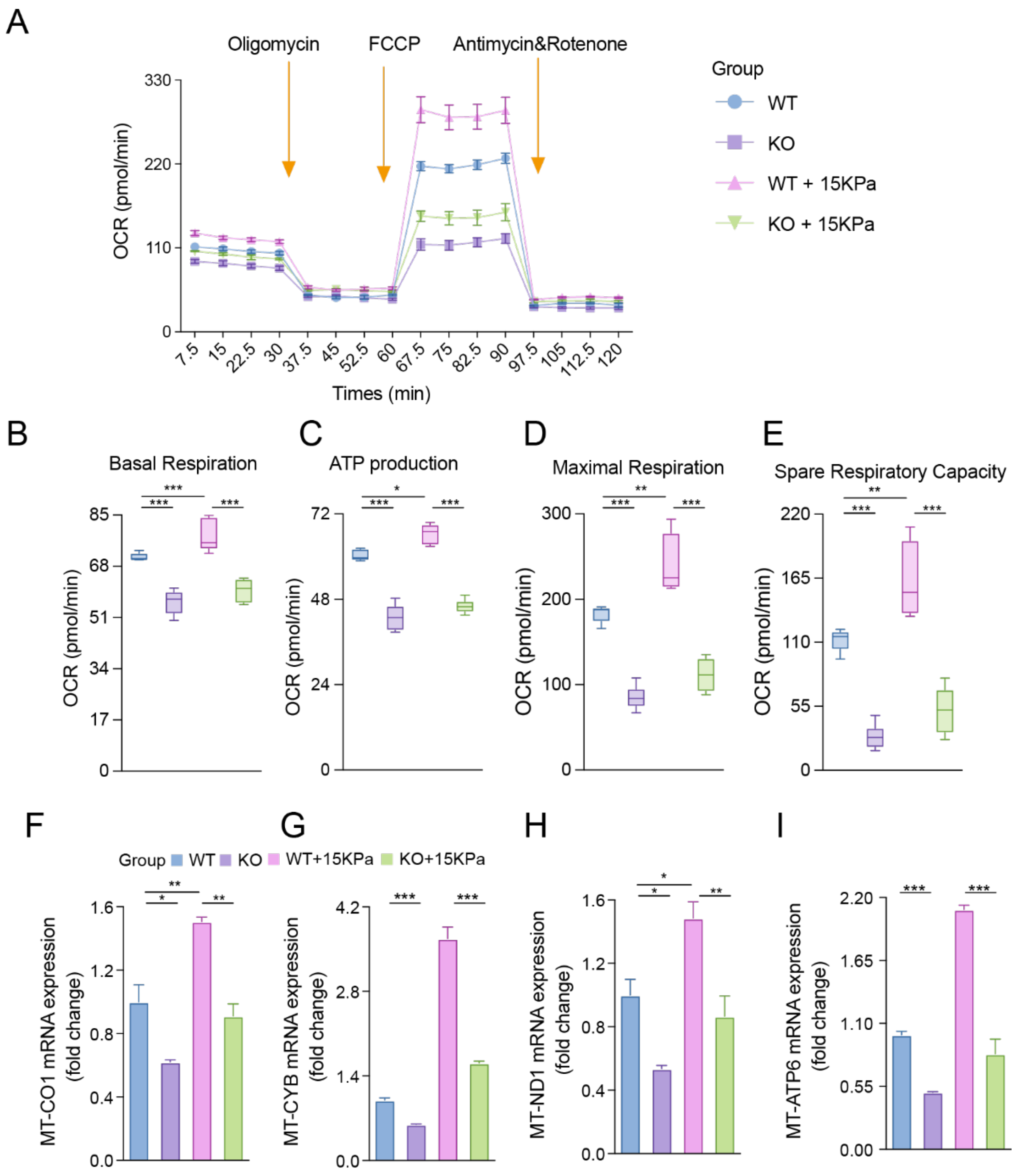

Figure S17. (A) Dynamic changes in oxidative phosphorylation were assessed in Usp26 knockout and WT chondrocytes, with or without exposure to 15 kPa compression for 4 hours, by measuring the oxygen consumption rate (OCR) using a Seahorse extracellular flux analyzer. Quantitative analysis of OCR parameters was performed, including basal respiration **(B)**, ATP production **(C)**, maximal respiration **(D)**, and spare respiratory capacity **(E)**. **(F-I)** The gene expression of MT-CO1, MT-

CYB, MT-ND1 and MT-ATP6 in *Usp26* knockout and WT chondrocytes, with or without exposure to 15 KPa compression for 4 hours. *$P<0.05$, **$P<0.01$, ***$P<0.001$. *P*-values were analyzed by one-way ANOVA.

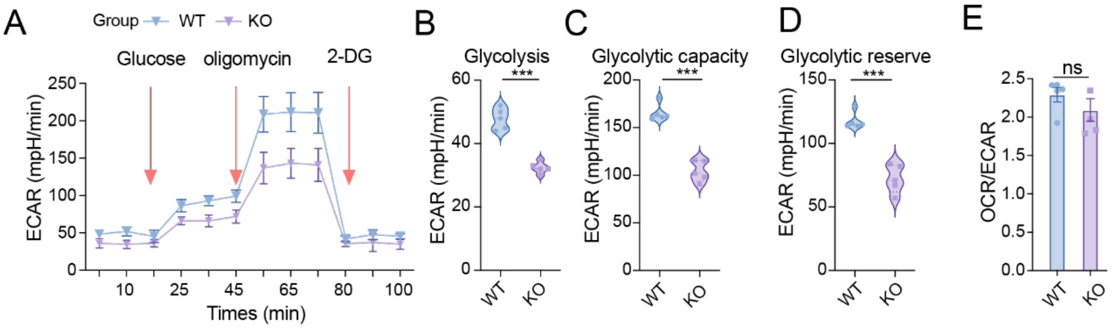

Figure S18. (A) Dynamic changes in glycolysis in Usp26 knockout chondrocytes and WT controls was assessed by measuring the ECAR, using a Seahorse extracellular flux analyzer. **(B-D)** Quantitative analysis of ECAR, including measurements of basal glycolysis (B), maximal glycolytic capacity (C), and glycolytic reserve (D). **(E)** The ratio of OCR to ECAR in WT and Usp26 knockout chondrocytes under basal conditions. ***$P<0.001$. *P*-values were analyzed by two-tailed *t* tests.

Table S1. Primer sequences for real time-PCR

**Gene**	**Forward primer**	**Reverse primer**	**Species**
Col10	AACAGGTATGCCCGTGTCTG	CCTACCCAAACGTGAGTCCC	Mice
Vegf	CTGCTGTACCTCCACCATGC	GTCTCAATCGGACGGCAGTA	Mice

Usp26	TGGTGTGGATGTTCGTGATCT	CCACTCTAGGCCGTTCTCAATAC	Mice
Alp	TCATTCCCACGTTTTCACATTC	GTTGTTGTGAGCGTAATCTACC	Mice
Mmp13	AGTAGTTTCCAGCACCGAATTA	CACTAACCTGGTGTCCAATAGT	Mice
Runx2	CCTTCAAGGTTGTAGCCCTC	GGAGTAGTTCTCATCATTCCCG	Mice
MT-ND1	CGAGACCGGTTCGATTTTGC	ATCTCCCAAGCGAAGATGCC	Mice
MT-CYB	ACGCAAACGGAGCCTCAATA	CCTCATGGAAGGACGTAGCC	Mice
MT-CO1	TCGGAGCCCCAGATATAGCA	TTTCCGGCTAGAGGTGGGTA	Mice
MT-ATP6	GCAGTCCGGCTTACAGCTAA	GGTAGCTGTTGGTGGGCTAA	Mice
β-actin	GGAGGGGGTTGAGGTGTT	GTGTGCACTTTTATTGGTCTCAA	Mice
Usp26	GACCTGGTAAGGGTGGGAGT	TCTCCGCAAGTAAGTGTCAATTT	Human
β-actin	CACAGAGCCTCGCCTTTGC	AATCCTTCTGACCCATGCCC	Human

Table S2. Primer sequences for ChIP-PCR

**Forward primer**	**Reverse primer**	**Species**
TGGCCAACATGGTGAAAC	TCTTCGGACAGACTCTCACTC	Human
